# Supplementary material for: Interventions to reduce interpersonal stigma towards patients with a mental dysregulation for ambulance and emergency department healthcare professionals: review protocol for an integrative review
Source: BMJ Open. 2023 Nov 2;13(11):e072604. doi: 10.1136/bmjopen-2023-072604 (PMC10626855; doi:10.1136/bmjopen-2023-072604)
Supplement: Supplementary data [file bmjopen-2023-072604supp001.pdf]

Appendix II: Search Strategy

Date Search: 13th July 2023

Medline/Pubmed:

Search	Query
#1	Attitude of Health Personnel[Mesh] OR Social Stigma[Mesh] OR Professional Competence[Mesh] OR "Professional-Patient Relations"[Mesh] OR "Social Discrimination"[Mesh] OR Attitude*[tiab] OR Stigma[tiab] OR Competence[tiab] OR "Professional Patient Relation*"[tiab] OR Discrimination[tiab] OR Traumatization[tiab] OR Traumatisation[tiab] OR "Secondary Trauma*"[tiab]
#2	Emergency Nursing[Mesh] OR "Emergency Nurs*"[tiab] OR "Emergency Room Nurs*"[tiab] OR "Trauma Nursing"[tiab] OR Emergency Medical Services[Mesh] OR Hospital Emergency Service[Mesh] OR "Emergency Medical Service*"[tiab] OR "Emergency Care"[tiab] OR "Emergency Health Service*"[tiab] OR "Emergency Department*"[tiab] OR "Emergency Room*"[tiab] OR "Emergency Unit*"[tiab] OR "Emergency Ward*"[tiab] OR "Emergency Hospital*"[tiab] OR "Emergency Service*"[tiab] OR Paramedics[Mesh] OR Paramedic*[tiab] OR "Emergency Physician*"[tiab] OR Ambulance*[tiab] OR "First Responder*"[tiab]
#3	Mental Disorders[Mesh] OR "Mental Disorder*"[tiab] OR "Mental Health Issue*"[tiab] OR "Mental Illness*"[tiab] OR "Mental Health Presentation*"[tiab] OR "Mental Health Problem*"[tiab] OR "Psychiatric Disorder*"[tiab] OR "Psychiatric Disease*"[tiab] OR "Psychiatric Illness*"[tiab] OR "Psychiatric Condition*"[tiab] OR Psychophysiologic Disorders[Mesh] OR "Psychophysiologic Disorder*"[tiab] OR "Psychosomatic Disorder*"[tiab] OR Aggression[Mesh] OR Aggression*[tiab] OR Problem Behavior[Mesh] OR "Problem Behavior*"[tiab] OR "Behavioral Problem*"[tiab] OR "Disruptive Behavior*"[tiab] OR "Behavioral Disorder*"[tiab] OR "Psychologically disturbed"[tiab] OR Self-Injurious Behavior[Mesh] OR "Self Injur*"[tiab] OR "Self Harm"[tiab] OR "Self Destruct*"[tiab] OR "Self Mutilat*"[tiab] OR Suicide[tiab] OR Suicidal[tiab]
#4	Intervention*[tiab] OR Action*[tiab] OR Educat*[tiab] OR Knowledge[tiab] OR Consult*[tiab] OR Training[tiab] OR Course*[tiab] OR Treatment[tiab] OR Therap*[tiab] OR Medication[tiab] OR Protocol*[tiab] OR Instrument*[tiab] OR Tool*[tiab]
#5	#1 AND #2 AND #3 AND #4

CINAHL:

Search	Query
#1	MH (“Attitude of Health Personnel+” OR “Professional Competence+” OR Stigma OR “Professional-Patient Relations+” OR Discrimination+) OR TI (Attitude* OR Stigma OR Competence OR “Professional Patient Relation*”OR Discrimination OR Traumatization OR Traumatisation OR “Secondary Trauma*”) OR AB (Attitude* OR Stigma OR Competence OR “Professional Patient Relation*” OR Discrimination OR Traumatization OR Traumatisation OR “Secondary Trauma*”)
#2	MH (“Emergency Medical Services+” OR “Emergency Nursing+” OR “Emergency Medical Technicians”) OR TI (“Emergency Nurs*” OR “Emergency Room Nurs*” OR “Trauma Nursing” OR “Emergency Medical Service*” OR “Emergency Care” OR “Emergency Health Service*” OR “Emergency Department*” OR “Emergency Room*” OR “Emergency Unit*” OR “Emergency Ward*” OR “Emergency Hospital*” OR “Emergency Service*” OR Paramedic* OR “Emergency Physician*” OR Ambulance* OR “First Responder*”) OR AB (“Emergency Nurs*” OR “Emergency Room Nurs*” OR “Trauma Nursing” OR “Emergency Medical Service*” OR “Emergency Care” OR “Emergency Health Service*” OR “Emergency Department*” OR “Emergency Room*” OR “Emergency Unit*” OR “Emergency Ward*” OR “Emergency Hospital*” OR “Emergency Service*” OR Paramedic* OR “Emergency Physician*” OR Ambulance* OR “First Responder*”)
#3	(MH (“Mental Disorders+” OR “Suicide+” OR “Self-Injurious Behavior”) OR TI (“Mental Disorder*” OR “Mental Health Issue*” OR “Mental Illness*” OR “Mental Health Presentation*” OR “Psychiatric Disorder*” OR “Psychiatric Disease*” OR “Psychiatric Illness*” OR “Psychiatric Condition*” OR “Psychophysiologic Disorder*” OR “Psychosomatic Disorder*” OR Aggression* OR Aggressive OR “Problem Behavior*” OR “Behavioral Problem*” OR “Behavior Problem*” OR “Behavior Disorder*” OR “Disruptive Behavior*” OR “Behavioral Disorder*” OR “Psychologically disturbed” OR “Self Injur*” OR “Self Harm” OR “Self Destruct*” OR “Self Mutilat*” OR Suicide OR Suicidal) OR AB (“Mental Disorder*” OR “Mental Health Issue*” OR “Mental Illness*” OR “Mental Health Presentation*” OR “Psychiatric Disorder*” OR “Psychiatric Disease*” OR “Psychiatric Illness*” OR “Psychiatric Condition*” OR “Psychophysiologic Disorder*” OR “Psychosomatic Disorder*” OR Aggression* OR Aggressive OR “Problem Behavior*” OR “Behavioral Problem*” OR “Behavior Problem*” OR “Behavior Disorder*” OR “Psychologically disturbed” OR “Self Injur*” OR “Self Harm” OR “Self Destruct*” OR “Self Mutilat*” OR Suicide OR Suicidal)

#4	TI(Intervention* OR Action* OR Educat* OR Knowledge OR Consult* OR Training OR Course* OR Treatment OR Therap* OR Medication OR Protocol* OR Instrument* OR Tool*) OR AB(Intervention* OR Action* OR Educat* OR Knowledge OR Consult* OR Training OR Course* OR Treatment OR Therap* OR Medication OR Protocol* OR Instrument* OR Tool*)
#5	#1 AND #2 AND #3 AND #4

PsycINFO:

Search	Query
#1	DE (Stigma OR “Health Personnel Attitudes” OR “Professional Competence+” OR Discrimination+) OR TI (Attitude* OR Stigma OR Competence OR “Professional Patient Relation*” OR Discrimination OR Traumatization OR Traumatisation OR “Secondary Trauma*”) OR AB (Attitude* OR Stigma OR Competence OR “Professional Patient Relation*” OR Discrimination OR Traumatization OR Traumatisation OR “Secondary Trauma*”)
#2	DE (“Emergency Services+” OR “Emergency Personnel+”) OR TI (“Emergency Nurs*” OR “Emergency Room Nurs*” OR “Trauma Nursing” OR “Emergency Medical Service*” OR “Emergency Care” OR “Emergency Health Service*” OR “Emergency Department*” OR “Emergency Room*” OR “Emergency Unit*” OR “Emergency Ward*” OR “Emergency Hospital*” OR “Emergency Service*” OR Paramedic* OR “Emergency Physician*” OR Ambulance* OR “First Responder*”) OR AB (“Emergency Nurs*” OR “Emergency Room Nurs*” OR “Trauma Nursing” OR “Emergency Medical Service*” OR “Emergency Care” OR “Emergency Health Service*” OR “Emergency Department*” OR “Emergency Room*” OR “Emergency Unit*” OR “Emergency Ward*” OR “Emergency Hospital*” OR “Emergency Service*” OR Paramedic* OR “Emergency Physician*” OR Ambulance* OR “First Responder*”)
#3	((DE (“Mental Disorders+” OR “Behavior Disorders+” OR “Behavior Problems+” OR “Aggressive Behavior+” OR “Self-Destructive Behavior+”) OR TI (“Mental Disorder*” OR “Mental Health Issue*” OR “Mental Illness*” OR “Mental Health Presentation*” OR “Psychiatric Disorder*” OR “Psychiatric Disease*” OR “Psychiatric Illness*” OR “Psychophysiologic Disorder*” OR “Psychosomatic Disorder*” OR Aggression* OR Aggressive OR “Problem Behavior*” OR “Behavioral Problem*” OR “Behavior Problem*” OR “Behavior Disorder*” OR “Disruptive Behavior*” OR “Behavioral Disorder*” OR “Psychologically disturbed” OR “Self Injur*” OR “Self Harm” OR “Self Destruct*” OR “Self Mutilat*” OR Suicide OR Suicidal) OR AB (“Mental Disorder*” OR “Mental Health Issue*” OR “Mental Illness*” OR “Mental Health Presentation*” OR “Psychiatric Disorder*” OR “Psychiatric Disease*” OR “Psychiatric Illness*” OR

	“Psychophysiologic Disorder*” OR “Psychosomatic Disorder*” OR Aggression* OR Aggressive OR “Problem Behavior*” OR “Behavioral Problem*” OR “Behavior Problem*” OR “Behavior Disorder*” OR “Disruptive Behavior*” OR “Behavioral Disorder*” OR “Psychologically disturbed” OR “Self Injur*” OR “Self Harm” OR “Self Destruct*” OR “Self Mutilat*” OR Suicide OR Suicidal))
#4	TI(Intervention* OR Action* OR Educat* OR Knowledge OR Consult* OR Training OR Course* OR Treatment OR Therap* OR Medication OR Protocol* OR Instrument* OR Tool*) OR AB(Intervention* OR Action* OR Educat* OR Knowledge OR Consult* OR Training OR Course* OR Treatment OR Therap* OR Medication OR Protocol* OR Instrument* OR Tool*)
#5	#1 AND #2 AND #3 AND #4
