# Supplementary material for: Interventions to reduce interpersonal stigma towards patients with a mental dysregulation for ambulance and emergency department healthcare professionals: review protocol for an integrative review
Source: BMJ Open. 2023 Nov 2;13(11):e072604. doi: 10.1136/bmjopen-2023-072604 (PMC10626855; doi:10.1136/bmjopen-2023-072604)
Supplement: Supplementary data [file bmjopen-2023-072604supp002.pdf]

Appendix I: PRISMA-P checklist

Section and topic	Item No	Checklist item	How and where is the item addressed in this review protocol
**ADMINISTRATIVE INFORMATION**			
Title:			
Identification	1a	Identify the report as a protocol of a systematic review	the title (p1) and methods (p7) describe the review as an integrative review.
Update	1b	If the protocol is for an update of a previous systematic review, identify as such	Not applicable
Registration	2	If registered, provide the name of the registry (such as PROSPERO) and registration number	PROSPERO ID number: CRD42023390664 Added to the abstract (p2)
Authors:			
Contact	3a	Provide name, institutional affiliation, e-mail address of all protocol authors; provide physical mailing address of corresponding author	Name, affiliations of all protocol authors are listed. Email address and physical mailing address of the corresponding author is provided on the title page. E-mail addresses of co-authors are presented to the publisher of the review protocol.
Contributions	3b	Describe contributions of protocol authors and identify the guarantor of the review	GVdG, NG, WHWH developed the initial protocol: review questions, introduction and methods. NB, LS, FS, JC, MvV commented on the manuscript and re-wrote sections of the manuscript. All authors documented their approval of the final version of the review protocol before submission. As senior researcher and project-leader WHWH is the guarantor of the review and review protocol.<br>This information can be found in the 'Author Statement' (p10).
Amendments	4	If the protocol represents an amendment of a previously completed or published protocol, identify as such and list changes; otherwise, state plan for documenting important protocol amendments	n.a.
Support:			
Sources	5a	Indicate sources of financial or other support for the review	Provided in the section Funding Statement (p10-11)
Sponsor	5b	Provide name for the review funder and/or sponsor	Provided in the section Funding Statement (p10-11)
Role of sponsor or funder	5c	Describe roles of funder(s), sponsor(s), and/or institution(s), if any, in developing the protocol	The funders/institutions have no role in developing the protocol. The authors, though working for some of these institutions, work independently on this research.
**INTRODUCTION**			
Rationale	6	Describe the rationale for the review in the context of what is already known	Provided in the introduction (p3-5)

Objectives	7	Provide an explicit statement of the question(s) the review will address with reference to participants, interventions, comparators, and outcomes (PICO)	Participants/Interventions: Provided in the Aim/Concepts/Research questions/ Methods and Analyses sections (p5-7)<br>Comparators will be documented, dependent on what is given in original publications.<br>Outcome variables: Main outcome variables: 1)Level of Interpersonal Stigma and 2) Patient satisfaction. The secondary outcome variable looked at is: Level of knowledge on psychiatry (p9).
**METHODS**			
Eligibility criteria	8	Specify the study characteristics (such as PICO, study design, setting, time frame) and report characteristics (such as years considered, language, publication status) to be used as criteria for eligibility for the review	All addressed in the protocol, in the 'Study selection and sorting'- section (p8).
Information sources	9	Describe all intended information sources (such as electronic databases, contact with study authors, trial registers or other grey literature sources) with planned dates of coverage	Listed in the Methods section: Medline, PsychInfo, CINAHL (p7).<br>Grey literature: Search via Google and contacting Professional Organizations for National Guidelines on the topic, in English and Dutch speaking countries (p7).
Search strategy	10	Present draft of search strategy to be used for at least one electronic database, including planned limits, such that it could be repeated	The full search strategy is attached as Appendix II to this review protocol.
Study records:			
Data management	11a	Describe the mechanism(s) that will be used to manage records and data throughout the review	Refworks Proquest will be used for management of the records.<br>Rayyan will be used for independent selection of studies, by two independent reviewers. Atlas.ti.23 will be used for data-extraction (p7).
Selection process	11b	State the process that will be used for selecting studies (such as two independent reviewers) through each phase of the review (that is, screening, eligibility and inclusion in meta-analysis)	Addressed in the 'Study selection and sorting' section. All steps in screening, in- and exclusion process, data-extraction and appraisal is carried out by two independent reviewers (p8).
Data collection process	11c	Describe planned method of extracting data from reports (such as piloting forms, done independently, in duplicate), any processes for obtaining and confirming data from investigators	Addressed in the 'Data extraction, analysis, and synthesis' section. Data extraction to be performed by two independent reviewers. Using ATLAS.ti (p9).
Data items	12	List and define all variables for which data will be sought (such as PICO items, funding sources), any pre-planned data assumptions and simplifications	Addressed in the 'Concepts' section (p5-6).
Outcomes and prioritization	13	List and define all outcomes for which data will be sought, including prioritization of main and additional outcomes, with rationale	Provided in the 'Data extraction, analysis, and synthesis' section (p9).

Risk of bias in individual studies	14	Describe anticipated methods for assessing risk of bias of individual studies, including whether this will be done at the outcome or study level, or both; state how this information will be used in data synthesis	Addressed in the 'Quality appraisal' section. Each included publication will be rated using the MMAT by two independent reviewers (p8-9).
Data synthesis	15a	Describe criteria under which study data will be quantitatively synthesized	a. and b. Given the nature of the outcome variables, and the expected paucity in available original research, we expect not to be able to quantitatively synthesize results.
	15b	If data are appropriate for quantitative synthesis, describe planned summary measures, methods of handling data and methods of combining data from studies, including any planned exploration of consistency (such as I^2, Kendall's τ)	Included studies will be listed in tables, presenting the main characteristics of the included studies: type of innovation, level of development, outcome measures, type of evaluation/testing of the innovations.
	15c	Describe any proposed additional analyses (such as sensitivity or subgroup analyses, meta-regression)	For each of the included studies, the results of the quality appraisal, using the MMAT (Mixed Methods Appraisal Tool) will be documented.
	15d	If quantitative synthesis is not appropriate, describe the type of summary planned	
Meta-bias(es)	16	Specify any planned assessment of meta-bias(es) (such as publication bias across studies, selective reporting within studies)	Given the expected paucity in available original research we do not expect Meta-bias(es).
Confidence in cumulative evidence	17	Describe how the strength of the body of evidence will be assessed (such as GRADE)	We will use the MMAT results as an indication for the overall quality of included publications. If the number and quality of included publications exceeds expectations, the authors will decide on using GRADE as an instrument for grading the quality of the included research (p8-9).
