# Supplementary material for: Interventions to reduce interpersonal stigma towards patients with a mental dysregulation for ambulance and emergency department healthcare professionals: review protocol for an integrative review
Source: BMJ Open. 2023 Nov 2;13(11):e072604. doi: 10.1136/bmjopen-2023-072604 (PMC10626855; doi:10.1136/bmjopen-2023-072604)
Supplement: Author's manuscript [file bmjopen-2023-072604.draft_revisions.pdf]

BMJ Open

BMJ Open is committed to open peer review. As part of this commitment we make the peer review history of every article we publish publicly available.

When an article is published we post the peer reviewers' comments and the authors' responses online. We also post the versions of the paper that were used during peer review. These are the versions that the peer review comments apply to.

The versions of the paper that follow are the versions that were submitted during the peer review process. They are not the versions of record or the final published versions. They should not be cited or distributed as the published version of this manuscript.

BMJ Open is an open access journal and the full, final, typeset and author-corrected version of record of the manuscript is available on our site with no access controls, subscription charges or pay-per-view fees (http://bmjopen.bmj.com).

If you have any questions on BMJ Open's open peer review process please email info.bmjopen@bmj.com

Review Protocol for an Integrative Review on Interventions to reduce Interpersonal Stigma towards patients with a mental dysregulation for ambulance and emergency department healthcare professionals

Journal:	*BMJ Open*
Manuscript ID	bmjopen-2023-072604
Article Type:	Protocol
Date Submitted by the Author:	08-Feb-2023

Complete List of Authors:	Van de Glind, Geurt; Hogeschool Utrecht, Institute of Nursing Studies<br>Galenkamp, Niek; Hogeschool Utrecht, Institute of Nursing Studies<br>Bleijenberg, Nienke; Hogeschool Utrecht<br>Schoonhoven, Lisette; University Medical Center Utrecht; University of Southampton<br>Scheepers, Floortje E; University Medical Centre Utrecht Brain Centre; University of Southampton<br>Crilly, Julia; Menzies Health Institute Queensland, Griffith University, Alliance for Vascular Access Teaching and Research, NHMRC Centre of Research Excellence in Nursing (NCREN); Gold Coast Health, Department of Emergency Medicine<br>van Veen, Mark; Hogeschool Utrecht, Institute of Nursing Studies<br>Ham, Wietske H.W.; Hogeschool Utrecht, Institute of Nursing Studies

Keywords:	PSYCHIATRY, ACCIDENT & EMERGENCY MEDICINE, MENTAL HEALTH

SCHOLARONE™ Manuscripts

Title

Review Protocol for an Integrative Review on Interventions to reduce Interpersonal Stigma towards patients with a mental dysregulation for ambulance and emergency department healthcare professionals

Corresponding author: Geurt van de Glind

E-mail address of the corresponding author: geurt.vandeglind@hu.nl

Physical mailing address of the corresponding author:

University of Applied Sciences Utrecht

PO box 12011

3501 AA Utrecht

The Netherlands

For peer review only

Authors

Geurt Van de Glind , Niek Galenkamp , Nienke Bleijenberg , Lisette Schoonhoven[2,3], Floortje E. Scheepers[4], Julia Crilly[5, 6], Mark van Veen , Wietske H.W. Ham

University of Applied Sciences, Utrecht, the Netherlands

[2] Nursing Science, Julius Center for Health Sciences and Primary Care, University Medical Center Utrecht/ Utrecht University, Utrecht, The Netherlands;

[3] School of Health Sciences, Faculty of Environmental and Life Sciences, University of Southampton, UK.

[4] University Medical Center Utrecht, The Netherlands

[5] Department of Emergency Medicine, Gold Coast Health, Gold Coast, Queensland, Australia

[6] School of Nursing and Midwifery/ Menzies Health Institute Queensland/ Centre for Mental Health, Griffith University, Gold Coast, Queensland, Australia

Key Words

Emergency Care, Social Stigma, Psychiatry

Word count:

Excluding ADDITIONAL INFORMATION: 2239 words

Including ADDITIONAL INFORMATION: 2532 words

ABSTRACT

Introduction: Worldwide, there is a constant increase of the extent and severity of mental illness. An exacerbation of (underlying) somatic complaints in this group of people can result in (recurring) ambulance and emergency department care - the emergency care context. The care for patients with a mental dysregulation seeking emergency care can be complex, intensive and comprises several challenges, possibly evoking a wide variety of feelings, ranging from worry or pity to annoyance and frustration in emergency care staff members. These challenges and feelings may lead to stigma towards patients with a mental dysregulation who seek emergency care. Various interventions have been developed impacting attitude and behavior and minimizing stigma held by health care professionals. However, these interventions are not explicitly aimed at the emergency care context nor do these represent the perspectives of the healthcare professionals that deliver the required emergency care. Therefore, the aim of the proposed review is to gain insight into interventions targeting healthcare professionals, that minimize stigma including beliefs, attitudes, and behavior towards patients with a mental dysregulation within the emergency care context.

Methods and analysis*:* The protocol for a systematic integrative review is presented, using the PRISMA-P recommendations for systematic reviews. Study selection and data-extraction will be performed by two independent reviewers. In each of the steps, an expert with lived experience will comment on both process and results. Software applications like Refworks-proquest, Rayyan and ATLAS.ti will be used to enhance the quality of the review, efficiency of the research work, and transparency of process and results.

Registration*:* The proposed review is registered at PROSPERO (https://www.crd.york.ac.uk/prospero/) under number CRD42023390664

For peer review only

INTRODUCTION

Worldwide, there is a constant increase in the extent and severity of mental illness (1,2), exacerbated by the COVID-19 pandemic (3,4). More than a quarter (5) or even half (6) of the world's population experience an episode with severe mental problems at some point during their life course. The burden of mental illness both on a socio-economic level and on the quality of life is immense (1,2,5,6). The World Health Organization (WHO) indicates that patients with chronic mental illness are 40-60% more likely to die 10 to 30 years earlier compared to people without a mental illness (5). People with mental illnesses have an increased risk for other somatic chronic illnesses (7-9). Furthermore, for these people maintaining a healthy lifestyle, financial stability and access to appropriate somatic care are often problematic (10). Apart from (acute situations due to) their

mental problems, an exacerbation of (underlying) somatic complaints in this group of people can result in (recurring) ambulance and emergency department care - the emergency care context (7,9,11-13).

To describe patients with mental problems who seek emergency care, several definitions or terms are used in the scientific literature. These include patients with 'mental illness' (14), 'mental disorder (15)' or 'mental health crisis' (16). Other authors focus on a specific selection of patients with mental problems that need emergency care, such as patients with 'borderline personality disorders' (17), 'substance use disorders' (18) or 'suicide attempts' (19). The problem with these descriptions is that uniformity is lacking and these do not fit in with all enrolled patients in acute healthcare. Moreover, their healthcare conditions (such as mental health disorders) are in many cases initially unknown and uncharted. This adds to the reasons why emergency care professionals typically focus on vital signs and behaviors in order to chart and treat acute physical healthcare problems. Therefore, the authors of this review protocol propose the concept 'mental dysregulation' for use in researching care for this vulnerable group in emergency care. The authors propose the following definition: '*In the emergency care context, patients with mental problems that seek emergency care may experience disproportionate feelings like fear, anger, sadness, or confusion, possibly with associated behaviors. When the aforementioned symptoms interfere with the patient's treatment or that of others, the patient is considered to have a mental dysregulation'*. This concept and definition will be subject to a Delphi-procedure including international experts on this topic in 2023-2024 for further harmonizing concepts used in international research on this matter.

The care for patients with a mental dysregulation who seek emergency care can be complex and intensive. In addition to a physical disorder or illness, these patients may be confused or emotionally unstable (9,10,20-24), which may hinder the identification and treatment of both physical and mental health symptoms. The provision of care for patients with a mental dysregulation can evoke a wide variety of feelings in acute healthcare providers, ranging from worry or pity, to annoyance and frustration (9,25-28). These feelings may originate from several challenges in the emergency care for these patients. First, these challenges may be induced by the still existing and experienced segregation between somatic and mental treatment, long waiting times (29), and revisits of patients with a mental dysregulation which cause irritation and a decrease in confidence in the effectiveness of interventions. Second, healthcare professionals in the emergency care context do not always have the right knowledge of psychopathology and legislation (22,30) or the skills to (timely) use de-escalation techniques (31,32), which can lead to frustration.

These challenges and feelings may lead to stigma towards patients with a mental dysregulation who seek emergency care (27-30,32,33). Several qualitative studies have revealed that patients with

For peer review only

mental problems often feel stigmatized by healthcare professionals in the emergency care context (16,33-35). A recent scoping review of factors that influence emergency department nurses care for patients with mental illness revealed that the emergency department environment greatly impacts the role of emergency department nurses, that their beliefs and perceptions leads to role conflicts, and the lack of knowledge and confidence impacts their view towards patients with mental illness. Specifically, some nurses reported they avoided patients, judged them as less important and attention seeking(26), which implies stigma towards these group of patients.

In their landmark paper Link & Phelan (2001) define stigma as existing when elements of labeling, stereotyping, separating ("us" from "them"), status loss, and discrimination co-occur in a power situation that allows these processes to unfold (36). Thornicroft et al. (2022) use this definition and elaborate on four different types of stigma of which, for this review, interpersonal stigma (also referred to as 'public stigma') is important. Interpersonal stigma refers to the link between stereotypes, negative attitudes (prejudice), and negative behavior (discrimination) towards people with mental health conditions. Three related components are part of interpersonal stigma: knowledge (ignorance or misinformation), attitudes (negative emotional reactions, such as prejudice) and behavior (such as avoidance or rejection) (37).

Various interventions have been developed that impact attitude and behavior and minimize stigma held by health care professionals. Thornicroft et al. (2022) report on 56 reviews, documenting changes in knowledge, attitudes, and clinical skills as well as clinical confidence and self-efficacy as stigma related outcomes. Several types of interventions are mentioned, such as e-interventions, use of simulations (e.g., standardized role-plays with actors), and social contact, such as filmed or live recovery testimonials. Studies involving different methods of contact (e.g., live or filmed) were more often associated with better outcomes for knowledge and attitudes when compared to educational interventions alone. Interventions including experts with lived experience in the design and evaluation of stigma interventions demonstrated better outcomes (37). The interventions outlined from the reviews covered various populations (e.g., mental health professionals, health care providers) and various mental health problems (e.g., dementia care, suicide prevention, borderline personality disorder); only one publication was specific to emergency department healthcare staff and focused on their attitudes towards patients with substance-related presentations (38). To our knowledge, a comprehensive review of the available scientific evidence in this specific context (ambulance and emergency department) is lacking.

For peer review only

AIM

To review the body of evidence to gain insight into interventions targeting healthcare professionals, that minimize stigma including beliefs, attitudes, and behavior towards patients with a mental dysregulation within the emergency care context.

CONCEPTS

For this review, we define *patients with a mental dysregulation* in the emergency care context as follows: patients with mental problems that seek emergency care may experience disproportionate feelings like fear, anger, sadness, or confusion, possibly with associated behaviors. When the aforementioned symptoms interfere with the patient's treatment or that of others, the patient is considered to have a mental dysregulation.

We applied the World Health Organization definition of '*Intervention*', namely, 'an act performed for, with or on behalf of a person or population whose purpose is to assess, improve, maintain, promote, or modify health, functioning or health conditions' (39).

Stigma is defined as the link between stereotypes, negative attitudes, and discrimination against people with mental health conditions in society (37).

The emergency care context in this review is defined as emergency care from the scene of acute incident of illness, followed by ambulance transport to and treatment in the emergency department.

Healthcare professionals include paramedics, emergency care nurses, nurse specialists, physicians and physician assistants that are directly involved in providing emergency care.

RESEARCH QUESTIONS

1. Which interventions that address Interpersonal Stigma towards patients with a mental dysregulation have been designed, implemented and evaluated for healthcare professionals in the emergency context?
2. From the healthcare professionals' perspective, which facilitators and barriers were encountered when implementing these interventions within the emergency care context?
3. From the service users' perspective, which facilitators and barriers were encountered when implementing these interventions within the emergency care context?

For peer review only

METHODS AND ANALYSIS

We will perform an integrative review of empirical literature using methodological strategies proposed by Whittemore and Knafl (2005) (40), using a 7-step framework outlined by Dhollande et al., (2021) (41) to identify current state of the art knowledge on the topic and to identify current knowledge gaps in research. The 7-steps applied to our integrative review will involve: 1) write the review question; 2) determine the search strategy; 3) critically appraise the search results; 4) summarize the search results; 5) extract and reduce data; 6) analysis; and 7) conclusions and implications (41)An integrative review allows for inclusion of diverse study methodologies, including qualitative, quantitative, experimental, and mixed-method studies, which makes it suitable for topics that have not been studied intensively. Results from an integrative review provide a complete and varied perspective of the studied topic.

To further enhance the quality of the review, the PRISMA-P recommendations will be followed where applicable (Shamseer et al., 2015) (42). The PRISMA-P checklist is attached as Appendix I.

Patient and Public Involvement

As Thornicroft and colleagues highlight in their publication on stigma it is important to involve people with lived experience of mental health conditions (PWLE) in both research and innovation projects (37). For that reason one or two PWLE will be included in the project team and co-author the review. For the development of this review protocol no patient or PWLE was involved.

Data sources and search strategy

A systematic search will be applied, followed by identifying possible missed publications via the reference lists of included publications. The following scientific databases will be systematically searched for English and Dutch publications: CINAHL, PsychINFO and MEDLINE. Search terms will include ‘Stigma’, Interpersonal stigma’, ‘Public stigma’, Interventions, Emergency Healthcare’, ‘Emergency Healthcare staff’, 'mental health', emergency department', 'ambulance', 'paramedics' and related search terms, as used in the forementioned databases. The full search strategy is attached as Appendix II. The results will be presented in the review. Additionally, we will search the grey literature. We will limit this grey literature to National Guidelines related to this topic (Acute Healthcare, Acute Psychiatric Care) in English or Dutch. We will search via Google and via Guideline Organizations (e.g., NICE in the United Kingdom) and professional organizations (e.g., the American Academy of Emergency Medicine).

For peer review only

Study selection and sorting

Studies will be included using the following inclusion and exclusion criteria:

Inclusion

- Original research report, both qualitative and quantitative studies;
- RCTs, prospective and retrospective cohort studies, case control studies, cross sectional studies and, if available, literature reviews;
- Description of innovation projects related to the topic; depending on information on outcome variables (see *Data extraction, analysis, and synthesis*-paragraph below);
- Focused on intervention(s), targeting Healthcare Professionals, to reduce stigma on mental health problems held by emergency care staff;
- Peer reviewed publication;
- Published in English or Dutch.

Exclusion

- Abstract and/ or full text not available

Study-selection: The results of the search will be assessed in the following steps: 1) Review of title and abstract and 2) Full text review of selected publications. Publications that clearly do not answer the research question nor addressing the inclusion criteria will be excluded (i.e., other topic, not an emergency care context, no stigma related intervention, language). Both steps will be performed by two independent reviewers, using Rayyan, a web-based tool, designed for publications-selection in scientific literature review (43). When no consensus is reached on a particular publication a third reviewer will make a final decision. – A PRISMA flow diagram will be used to provide a summary of the search and selection process.

Quality appraisal

The quality of included studies will be assessed with the Mixed Methods Appraisal Tool (44), developed and improved by Hong et al., (2018) (45). Each included publication will be assessed by two independent reviewers. Quality appraisal is not used for study selection, but for means of interpreting the strength of findings and conclusions. As Thornicrofft et al., (2022) (37) emphasize the importance of including persons with lived experience in the development of interventions, we will include this in reviewing and appraising interventions presented in the included publications, by

For peer review only

answering this question: To what extent are people with lived experience involved in the design/ delivery/ evaluation of the intervention and if involved, what were the results of this?

Data extraction, analysis, and synthesis

Data extraction will be performed using Atlas.ti, a software application, originally designed for qualitative research analyses purposes, but also used for transparent data extraction and analyses in literature review (46). A predetermined set of labels will be set up within ATLAS.ti and will include (but is not limited to): definition of stigma, description of intervention, study population, study design, sample, site/setting, measurements, outcome variable(s), reported strengths of the study, reported limitations of the study.

The main outcome variables of interest are 1) Level of Interpersonal Stigma and 2) Patient satisfaction. The secondary outcome variable of interest is: Level of knowledge on psychiatry.

Depending on the instruments used in the original publications, the measures and instruments for results on the outcome variables will be listed, when appropriate in table(s). In the discussion part of the review, the quality of the used instruments will be discussed.

Two independent reviewers will analyze the included publications, identifying (by adding the predefined labels to) citations in the publications, relevant for answering the review questions. Depending on the content of the results, we will, when appropriate, perform thematic analysis as described by Dhollande et al., (2021) (41).

The selected citations will be further analyzed and discussed by the authors, using ATLAS.ti tools, whereafter the extracted data will be used for answering the research questions.

Strength and limitations

The proposed review will contain a discussion and conclusions section. Within the discussion section, strength and limitations of the review will be discussed.

ADDITIONAL INFORMATION

Planning and time-frame

Start search: Immediately after submission of the review protocol: February 2023.

For peer review only

Study selection: February/March 2023

Data extraction and synthesis: March/May 2023

Writing and submission of the review: May/August 2023

Ethics and dissemination

No ethical approval or safety considerations are required for this review. The proposed review will be submitted to a relevant peer reviewed international journal. Results will be presented at relevant medical scientific conferences.

Author Statement

GVdG, NG, WHWH developed the initial protocol: review questions, introduction and methods. NB, LS, FS, JC and MvV commented on the manuscript and re-wrote sections of the manuscript. All authors documented their approval of the final version of the review protocol before submission. As senior researcher and project-leader WHWH is the guarantor of the review and review protocol.

Data Statement

As ATLAS.ti is used for data extraction, analysis and synthesis, used citations including used labels from included publications are available via the corresponding author.

Declaration of Conflicting Interests

The authors declare to have no conflicts of interest related to this review.

Funding statement

This review is part of the PONTAC-study (Psychisch ONTregeld in de Acute zorg/ Mental dysregulation in Acute healthcare). The PONTAC study is co-financed by SIA-RAAK, part of the Netherlands Organization for Scientific Research (NWO), under award/grant number: RAAK.PUB08.029. Furthermore, this study was partly funded by a fellowship funding from the Netherlands Organisation for Health Research and Development (ZonMw), under award/grant number number 10040022110004, was awarded to dr. Wietske H.W. Ham (Wietske Blom-Ham).

For peer review only

The University of Applied Sciences, Utrecht, University Medical Centre, Utrecht, Diakonessenhuis, Utrecht, St.Antionius Hospital, Utrecht, Meander Medical Center, Amersfoort, Regional Ambulance Service Utrecht, Altrecht, Mental Healthcare, Utrecht have contributed in-kind to the study. The latter 7 institutions did not provide an award/grant number to this project.

For peer review only

References

(1) Whiteford HA, Ferrari AJ, Degenhardt L, Feigin V, Vos T. The global burden of mental, neurological and substance use disorders: an analysis from the Global Burden of Disease Study 2010. PLoS One 2015;10(2):e0116820.

(2) Arias D, Saxena S, Verguet S. Quantifying the global burden of mental disorders and their economic value. EClinicalMedicine 2022 Sep 28;54:101675.

(3) Boden M, Zimmerman L, Azevedo KJ, Ruzek JI, Gala S, Abdel Magid H,S., et al. Addressing the mental health impact of COVID-19 through population health; PMC7934657. Clin.Psychol.Rev. 2021;85(102006):102006.

(4) Xiong J, Lipsitz O, Nasri F, Lui LMW, Gill H, Phan L, et al. Impact of COVID-19 pandemic on mental health in the general population: A systematic review; PMC7413844. J.Affect.Disord. 2020;277:55-64.

(5) Kessler RC, Aguilar-Gaxiola S, Alonso J, Chatterji S, Lee S, Ormel J, et al. The global burden of mental disorders: an update from the WHO World Mental Health (WMH) surveys; PMC3039289. Epidemiol.Psichiatr.Soc. 2009;18(1):23-33.

(6) Organisation for Economic Co-operation and Development. OECD Mental Health. Available at: https://www.oecd.org/els/health-systems/mental-health.htm. Accessed 17th January, 2023.

(7) Scott KM, Lim C, Al-Hamzawi A, Alonso J, Bruffaerts R, Caldas-de-Almeida JM, et al. Association of mental disorders with subsequent chronic physical conditions: World mental health surveys from 17 countries; PMC5333921. JAMA Psychiatry 2016;73(2):150-158.

(8) Momen NC, Plana-Ripoll O, Agerbo E, Benros ME, Børglum A,D., Christensen MK, et al. Association between mental disorders and subsequent medical conditions; PMC7261506. N.Engl.J.Med. 2020;382(18):1721-1731.

(9) Giandinoto J, Edward K. Challenges in acute care of people with co-morbid mental illness. Br J Nurs 2014 Jul 10-23;23(13):728-732.

(10) Giandinoto J, Edward K. The phenomenon of co-morbid physical and mental illness in acute medical care: the lived experience of Australian health professionals. BMC Res Notes 2015 Jul 07;8:295.

(11) Theriault KM, Rosenheck RA, Rhee TG. Increasing Emergency Department Visits for Mental Health Conditions in the United States. J Clin Psychiatry 2020 Jul 28;81(5).

(12) Ronaldson A, Elton L, Jayakumar S, Jieman A, Halvorsrud K, Bhui K. Severe mental illness and health service utilisation for nonpsychiatric medical disorders: A systematic review and meta-analysis. PLoS Med 2020 09;17(9):e1003284.

(13) Liu NH, Daumit GL, Dua T, Aquila R, Charlson F, Cuijpers P, et al. Excess mortality in persons with severe mental disorders: a multilevel intervention framework and priorities for clinical practice, policy and research agendas; PMC5269481. World Psychiatry 2017;16(1):30-40.

For peer review only

(14) Azar KMJ, Petersen JP, Shen Z, Nasrallah C, Pesa J, LaMori J, et al. Serious Mental Illness and Health-Related Factors Associated with Regional Emergency Department Utilization. Popul Health Manag 2020 Dec;23(6):430-437.

(15) Armoon B, Cao Z, Grenier G, Meng X, Fleury M. Profiles of high emergency department users with mental disorders. Am J Emerg Med 2022 Apr;54:131-141.

(16) Sacre M, Albert R, Hoe J. What are the experiences and the perceptions of service users attending Emergency Department for a mental health crisis? A systematic review. Int.J.Ment.Health Nurs. 2022;31(2):400-423.

(17) Artioli M, Bougon E, Mathur A, Salles J. Factors associated with a borderline personality disorder diagnosis in the emergency department. Front Psychiatry 2022 Sep 28;13:925462.

(18) Dowdell EB, Alderman SE, Foushee N, Holland E, Reedy EA. Expressions of Compassion Fatigue by Emergency Department Nurses Caring for Patients With Opioid and Substance Use Disorders. J Emerg Nurs 2022 Nov;48(6):688-697.

(19) Rassy J, Daneau D, Larue C, Rahme E, Low N, Lamarre S, et al. Measuring Quality of Care Received by Suicide Attempters in the Emergency Department. Arch Suicide Res 2022;26(1):81-90.

(20) Innes K, Morphet J, O'Brien AP, Munro I. Caring for the mental illness patient in emergency departments--an exploration of the issues from a healthcare provider perspective. J Clin Nurs 2014 Jul;23(13-14):2003-2011.

(21) van Nieuwenhuizen A, Henderson C, Kassam A, Graham T, Murray J, Howard LM, et al. Emergency department staff views and experiences on diagnostic overshadowing related to people with mental illness. Epidemiol Psychiatr Sci 2013 Sep;22(3):255-262.

(22) Marynowski-Traczyk D, Moxham L, Broadbent M. Emergency Department Registered Nurses' conceptualisation of recovery for people experiencing mental illness. Australas Emerg Nurs J 2017 May;20(2):75-81.

(23) Rolfe U, Pope C, Crouch R. Paramedic performance when managing patients experiencing mental health issues - Exploring paramedics' Presentation of Self. Int Emerg Nurs 2020 03;49:100828.

(24) Shefer G, Henderson C, Howard LM, Murray J, Thornicroft G. Diagnostic overshadowing and other challenges involved in the diagnostic process of patients with mental illness who present in emergency departments with physical symptoms--a qualitative study. PLoS One 2014;9(11):e111682.

(25) Beks H, Healey C, Schlicht KG. 'When you're it': a qualitative study exploring the rural nurse experience of managing acute mental health presentations. Rural Remote Health 2018 08;18(3):4616.

(26) Perrone McIntosh J,T. Emergency department nurses' care of psychiatric patients: A scoping review. Int.Emerg.Nurs. 2021;54(100929):100929.

(27) Clarke D, Usick R, Sanderson A, Giles-Smith L, Baker J. Emergency department staff attitudes towards mental health consumers: a literature review and thematic content analysis. Int J Ment Health Nurs 2014 Jun;23(3):273-284.

For peer review only

(28) Emond K, O'Meara P, Bish M. Paramedic management of mental health related presentations: a scoping review. J Ment Health 2019 Feb;28(1):89-96.

(29) Sweeny A, Keijzers G, O'Dwyer J, Stapelberg NC, Crilly J. Patients with mental health conditions in the emergency department: Why so long a wait? Emerg Med Australas 2020 Jun 08,.

(30) Mérelle SYM, Boerema I, van der Linden MC, Gilissen R. [Issues in emergency care for people who attempted suicide]. Ned Tijdschr Geneeskd 2018 Jul 02,;162.

(31) Rolfe U, Pope C, Crouch R. Paramedic performance when managing patients experiencing mental health issues - Exploring paramedics' Presentation of Self. Int Emerg Nurs 2020 03;49:100828.

(32) McCann TV, Savic M, Ferguson N, Cheetham A, Witt K, Emond K, et al. Recognition of, and attitudes towards, people with depression and psychosis with/without alcohol and other drug problems: results from a national survey of Australian paramedics. BMJ Open 2018 12 04,;8(12):e023860.

(33) Wong AH, Ray JM, Rosenberg A, Crispino L, Parker J, McVaney C, et al. Experiences of Individuals Who Were Physically Restrained in the Emergency Department. JAMA Netw Open 2020 01 03,;3(1):e1919381.

(34) Daggenvoorde TH, Gijsman HJ, Goossens PJJ. Emergency care in case of acute psychotic and/or manic symptoms: Lived experiences of patients and their families with the first interventions of a mobile crisis team. A phenomenological study. Perspect Psychiatr Care 2018 Oct;54(4):462-468.

(35) Digel Vandyk A, Young L, MacPhee C, Gillis K. Exploring the Experiences of Persons Who Frequently Visit the Emergency Department for Mental Health-Related Reasons. Qual Health Res 2018 03;28(4):587-599.

(36) Link, Bruce G. Phelan, Jo C. Conceptualizing Stigma. annurev.soc. 2001;27:363-385.

(37) Thornicroft G, Sunkel C, Alikhon Aliev A, Baker S, Brohan E, El Chammay R, et al. The Lancet Commission on ending stigma and discrimination in mental health. Lancet 2022;400(10361):1438-1480.

(38) Gonzalez M, Clarke DE, Pereira A, Boyce-Gaudreau K, Waldman C, Demczuk L, et al. The impact of educational interventions on attitudes of emergency department staff towards patients with substance-related presentations: a quantitative systematic review. JBI Database System Rev Implement Rep 2017 Aug;15(8):2153-2181.

(39) Fortune N, Madden R, Almborg A. Use of a new international classification of Health Interventions for capturing information on Health Interventions relevant to people with disabilities; PMC5800244. Int.J.Environ.Res.Public Health 2018;15(1).

(40) Whittemore R, Knafl K. The integrative review: updated methodology. J.Adv.Nurs. 2005;52(5):546-553.

(41) Dhollande S, Taylor A, Meyer S, Scott M. Conducting integrative reviews: a guide for novice nursing researchers. J Res Nurs 2021 Aug;26(5):427-438.

For peer review only

(42) Shamseer L, Moher D, Clarke M, Ghersi D, Liberati A, Petticrew M, et al. Preferred reporting items for systematic review and meta-analysis protocols (PRISMA-P) 2015: elaboration and explanation. BMJ 2015 Jan 2;350:g7647.

(43) Rayyan - Faster systematic reviews. 2022; Available at: https://www.rayyan.ai/. Accessed 15 December, 2022.

(44) Mixed Methods Appraisal Tool - version 2018. Available at: http://mixedmethodsappraisaltoolpublic.pbworks.com/w/file/fetch/127916259/MMAT_2018_criteria-manual_2018-08-01_ENG.pdf. Accessed 20th December, 2022.

(45) Hong QN, Gonzalez-Reyes A, Pluye P. Improving the usefulness of a tool for appraising the quality of qualitative, quantitative and mixed methods studies, the Mixed Methods Appraisal Tool (MMAT). J.Eval.Clin.Pract. 2018;24(3):459-467.

(46) Smit B, Scherman V. Computer-Assisted Qualitative Data Analysis Software for Scoping Reviews: A Case of ATLAS.ti. International Journal of Qualitative Methods 2021;20:16094069211019140.

For peer review only

Appendix I: PRISMA-P checklist

Section and topic	Item No	Checklist item	How and where is the item addressed in this review protocol
**ADMINISTRATIVE INFORMATION**			
Title:			
Identification	1a	Identify the report as a protocol of a systematic review	the title and methods describe the review as a systematic integrative review.
Update	1b	If the protocol is for an update of a previous systematic review, identify as such	Not applicable
Registration	2	If registered, provide the name of the registry (such as PROSPERO) and registration number	PROSPERO ID number: CRD42023390664
Authors:			
Contact	3a	Provide name, institutional affiliation, e-mail address of all protocol authors; provide physical mailing address of corresponding author	Name, affiliations of all protocol authors are listed. Email address and physical mailing address of the corresponding author is provided on the title page. E-mail addresses of co-authors are presented to the publisher of the review protocol.
Contributions	3b	Describe contributions of protocol authors and identify the guarantor of the review	GVdG, NG, WHWH developed the initial protocol: review questions, introduction and methods. NB, LS, FS, JC, MvV commented on the manuscript and re-wrote sections of the manuscript. All authors documented their approval of the final version of the review protocol before submission. As senior researcher and project-leader WHWH is the guarantor of the review and review protocol.
Amendments	4	If the protocol represents an amendment of a previously completed or published protocol, identify as such and list changes; otherwise, state plan for documenting important protocol amendments	n.a.
Support:			
Sources	5a	Indicate sources of financial or other support for the review	Provided in the section 'Funding Statement'
Sponsor	5b	Provide name for the review funder and/or sponsor	Provided in the section 'Funding Statement'
Role of sponsor or funder	5c	Describe roles of funder(s), sponsor(s), and/or institution(s), if any, in developing the protocol	The funders/institutions have nor role in developing the protocol. The authors, though working for these institutions, work independently on this research.
**INTRODUCTION**			
Rationale	6	Describe the rationale for the review in the context of what is already known	Provided in the introduction
Objectives	7	Provide an explicit statement of the question(s) the review will address with reference to participants, interventions, comparators, and outcomes (PICO)	Participants/Interventions: Provided in the Concepts/Aim/Research questions/ Methods

For peer review only

			Comparators will be documented, dependent on what is given in original publications.<br>Outcome variables: Main outcome variables: 1)Level of Interpersonal Stigma and 2) Patient satisfaction. The secondary outcome variable looked at is: Level of knowledge on psychiatry.
**METHODS**			
Eligibility criteria	8	Specify the study characteristics (such as PICO, study design, setting, time frame) and report characteristics (such as years considered, language, publication status) to be used as criteria for eligibility for the review	All addressed in the protocol
Information sources	9	Describe all intended information sources (such as electronic databases, contact with study authors, trial registers or other grey literature sources) with planned dates of coverage	Listed in the Methods section: Medline, PsychInfo, CINAHL.<br>Grey literature: Search via Google and Professional Organizations for National Guidelines on the topic, in English and Dutch.
Search strategy	10	Present draft of search strategy to be used for at least one electronic database, including planned limits, such that it could be repeated	The full search strategy is attached as Appendix II to this review protocol.
Study records:			
Data management	11a	Describe the mechanism(s) that will be used to manage records and data throughout the review	Refworks Proquest will be used for management of the records.<br>Rayyan will be used for independent selection of studies, by two independent reviewers.
Selection process	11b	State the process that will be used for selecting studies (such as two independent reviewers) through each phase of the review (that is, screening, eligibility and inclusion in meta-analysis)	Addressed in the 'Study selection and sorting' section.
Data collection process	11c	Describe planned method of extracting data from reports (such as piloting forms, done independently, in duplicate), any processes for obtaining and confirming data from investigators	Addressed in the 'Data extraction, analysis, and synthesis' section. Data extraction to be performed by two independent reviewers. Using ATLAS.ti.
Data items	12	List and define all variables for which data will be sought (such as PICO items, funding sources), any pre-planned data assumptions and simplifications	Addressed in the 'Concepts' section.
Outcomes and prioritization	13	List and define all outcomes for which data will be sought, including prioritization of main and additional outcomes, with rationale	Provided in the 'Data extraction, analysis, and synthesis' section.
Risk of bias in individual studies	14	Describe anticipated methods for assessing risk of bias of individual studies, including whether this will be done at the outcome or study level, or both; state how this information will be used in data synthesis	Addressed in the 'Quality appraisal' section. Each included publication will be rated using the MMAT by two independent reviewers.

For peer review only

Data synthesis	15a	Describe criteria under which study data will be quantitatively synthesized	Given the nature of the outcome variables, and the expected paucity in available original research, we expect not to be able to quantitatively synthesize results.
	15b	If data are appropriate for quantitative synthesis, describe planned summary measures, methods of handling data and methods of combining data from studies, including any planned exploration of consistency (such as I^2, Kendall's τ)	
	15c	Describe any proposed additional analyses (such as sensitivity or subgroup analyses, meta-regression)	
	15d	If quantitative synthesis is not appropriate, describe the type of summary planned	
Meta-bias(es)	16	Specify any planned assessment of meta-bias(es) (such as publication bias across studies, selective reporting within studies)	Given the expected paucity in available original research we do not expect Meta-bias(es).
Confidence in cumulative evidence	17	Describe how the strength of the body of evidence will be assessed (such as GRADE)	We will use the MMAT results as an indication for the overall quality of included publications. If the number and quality of included publications exceeds expectations, the authors will decide on using GRADE as an instrument for grading the quality of the included research.

For peer review only

Appendix II: Search Strategy

Pubmed:

(Attitude of Health Personnel[Mesh] OR Social Stigma[Mesh] OR Professional Competence[Mesh] OR "Professional-Patient Relations"[Mesh] OR "Social Discrimination"[Mesh] OR Attitude*[tiab] OR Stigma[tiab] OR Competence[tiab] OR "Professional Patient Relation*"[tiab] OR Discrimination[tiab] OR Traumatization[tiab] OR Traumatisation[tiab] OR "Secondary Trauma*"[tiab])

(Emergency Nursing[Mesh] OR "Emergency Nurs*"[tiab] OR "Emergency Room Nurs*"[tiab] OR "Trauma Nursing"[tiab] OR Emergency Medical Services[Mesh] OR Hospital Emergency Service[Mesh] OR "Emergency Medical Service*"[tiab] OR "Emergency Care"[tiab] OR "Emergency Health Service*"[tiab] OR "Emergency Department*"[tiab] OR "Emergency Room*"[tiab] OR "Emergency Unit*"[tiab] OR "Emergency Ward*"[tiab] OR "Emergency Hospital*"[tiab] OR "Emergency Service*"[tiab] OR Paramedics[Mesh] OR Paramedic*[tiab] OR "Emergency Physician*"[tiab] OR Ambulance*[tiab])

(Mental Disorders[Mesh] OR "Mental Disorder*"[tiab] OR "Mental Health Issue*"[tiab] OR "Mental Illness*"[tiab] OR "Mental Health Presentation*"[tiab] OR "Mental Health Problem*"[tiab] OR "Psychiatric Disorder*"[tiab] OR "Psychiatric Disease*"[tiab] OR "Psychiatric Illness*"[tiab] OR "Psychiatric Condition*"[tiab] OR Psychophysiologic Disorders[Mesh] OR "Psychophysiologic Disorder*"[tiab] OR "Psychosomatic Disorder*"[tiab] OR Aggression[Mesh] OR Aggression*[tiab] OR Problem Behavior[Mesh] OR "Problem Behavior*"[tiab] OR "Behavioral Problem*"[tiab] OR "Disruptive Behavior*"[tiab] OR "Behavioral Disorder*"[tiab] OR "Psychologically disturbed"[tiab]) AND (Patients[Mesh] OR Patient*[tiab] OR Client*[tiab])

Intervention*[tiab] OR Action*[tiab] OR Educat*[tiab] OR Knowledge[tiab] OR Consult*[tiab] OR Training[tiab] OR Course*[tiab] OR Treatment[tiab] OR Therap*[tiab] OR Medication[tiab] OR Protocol*[tiab] OR Instrument*[tiab] OR Tool*[tiab]

CINAHL:

(MH ("Attitude of Health Personnel+" OR "Professional Competence+" OR Stigma OR "Professional-Patient Relations+" OR Discrimination+) OR TI (Attitude* OR Stigma OR Competence OR "Professional Patient Relation*"OR Discrimination OR Traumatization OR Traumatisation OR "Secondary Trauma*") OR AB (Attitude* OR Stigma OR Competence OR "Professional Patient Relation*" OR Discrimination OR Traumatization OR Traumatisation OR "Secondary Trauma*"))

(MH ("Emergency Medical Services+" OR "Emergency Nursing+" OR "Emergency Medical Technicians") OR TI ("Emergency Nurs*" OR "Emergency Room Nurs*" OR "Trauma Nursing" OR "Emergency Medical Service*" OR "Emergency Care" OR "Emergency Health Service*" OR "Emergency Department*" OR "Emergency Room*" OR "Emergency Unit*" OR "Emergency Ward*" OR "Emergency Hospital*" OR "Emergency Service*" OR Paramedic* OR "Emergency Physician*" OR Ambulance*) OR AB ("Emergency Nurs*" OR "Emergency Room Nurs*" OR "Trauma Nursing" OR "Emergency Medical Service*" OR "Emergency Care" OR "Emergency Health Service*" OR

For peer review only

"Emergency Department*" OR "Emergency Room*" OR "Emergency Unit*" OR "Emergency Ward*" OR "Emergency Hospital*" OR "Emergency Service*" OR Paramedic* OR "Emergency Physician*" OR Ambulance*))

((MH "Mental Disorders+" OR TI ("Mental Disorder*" OR "Mental Health Issue*" OR "Mental Illness*" OR "Mental Health Presentation*" OR "Psychiatric Disorder*" OR "Psychiatric Disease*" OR "Psychiatric Illness*" OR "Psychiatric Condition*" OR "Psychophysiologic Disorder*" OR "Psychosomatic Disorder*" OR Aggression* OR Aggressive OR "Problem Behavior*" OR "Behavioral Problem*" OR "Behavior Problem*" OR "Behavior Disorder*" OR "Disruptive Behavior*" OR "Behavioral Disorder*" OR "Psychologically disturbed") OR AB ("Mental Disorder*" OR "Mental Health Issue*" OR "Mental Illness*" OR "Mental Health Presentation*" OR "Psychiatric Disorder*" OR "Psychiatric Disease*" OR "Psychiatric Illness*" OR "Psychiatric Condition*" OR "Psychophysiologic Disorder*" OR "Psychosomatic Disorder*" OR Aggression* OR Aggressive OR "Problem Behavior*" OR "Behavioral Problem*" OR "Behavior Problem*" OR "Behavior Disorder*" OR "Psychologically disturbed")) AND (MH Patients+ OR TI (Patient* OR Client*) OR AB (Patient* OR Client*)))

(TI(Intervention* OR Action* OR Educat* OR Knowledge OR Consult* OR Training OR Course* OR Treatment OR Therap* OR Medication OR Protocol* OR Instrument* OR Tool*) OR AB(Intervention* OR Action* OR Educat* OR Knowledge OR Consult* OR Training OR Course* OR Treatment OR Therap* OR Medication OR Protocol* OR Instrument* OR Tool*))

<u>Psycinfo</u>:

(DE (Stigma OR "Health Personnel Attitudes" OR "Professional Competence+" OR Discrimination+) OR TI (Attitude* OR Stigma OR Competence OR "Professional Patient Relation*" OR Discrimination OR Traumatization OR Traumatisation OR "Secondary Trauma*") OR AB (Attitude* OR Stigma OR Competence OR "Professional Patient Relation*" OR Discrimination OR Traumatization OR Traumatisation OR "Secondary Trauma*"))

(DE ("Emergency Services+" OR "Emergency Personnel+") OR TI ("Emergency Nurs*" OR "Emergency Room Nurs*" OR "Trauma Nursing" OR "Emergency Medical Service*" OR "Emergency Care" OR "Emergency Health Service*" OR "Emergency Department*" OR "Emergency Room*" OR "Emergency Unit*" OR "Emergency Ward*" OR "Emergency Hospital*" OR "Emergency Service*" OR Paramedic* OR "Emergency Physician*" OR Ambulance*) OR AB ("Emergency Nurs*" OR "Emergency Room Nurs*" OR "Trauma Nursing" OR "Emergency Medical Service*" OR "Emergency Care" OR "Emergency Health Service*" OR "Emergency Department*" OR "Emergency Room*" OR "Emergency Unit*" OR "Emergency Ward*" OR "Emergency Hospital*" OR "Emergency Service*" OR Paramedic* OR "Emergency Physician*" OR Ambulance*))

((DE ("Mental Disorders+" OR "Behavior Disorders+" OR "Behavior Problems+" OR "Aggressive Behavior+") OR TI ("Mental Disorder*" OR "Mental Health Issue*" OR "Mental Illness*" OR "Mental Health Presentation*" OR "Psychiatric Disorder*" OR "Psychiatric Disease*" OR "Psychiatric Illness*" OR "Psychophysiologic Disorder*" OR "Psychosomatic Disorder*" OR Aggression* OR Aggressive OR "Problem Behavior*" OR "Behavioral Problem*" OR "Behavior Problem*" OR "Behavior Disorder*" OR "Disruptive Behavior*" OR "Behavioral Disorder*" OR "Psychologically disturbed") OR AB ("Mental Disorder*" OR "Mental Health Issue*" OR "Mental Illness*" OR "Mental Health Presentation*" OR "Psychiatric Disorder*" OR "Psychiatric Disease*" OR "Psychiatric Illness*" OR "Psychophysiologic Disorder*" OR "Psychosomatic Disorder*" OR Aggression* OR Aggressive OR

For peer review only

“Problem Behavior*” OR “Behavioral Problem*” OR “Behavior Problem*” OR “Behavior Disorder*” OR “Disruptive Behavior*” OR “Behavioral Disorder*” OR “Psychologically disturbed”)) AND (DE Patients+ OR TI (Patient* OR Client*) OR AB (Patient* OR Client*)))

(TI(Intervention* OR Action* OR Educat* OR Knowledge OR Consult* OR Training OR Course* OR Treatment OR Therap* OR Medication OR Protocol* OR Instrument* OR Tool*) OR AB(Intervention* OR Action* OR Educat* OR Knowledge OR Consult* OR Training OR Course* OR Treatment OR Therap* OR Medication OR Protocol* OR Instrument* OR Tool*))

For peer review only

BMJ Open

Review protocol for an integrative review on interventions to reduce interpersonal stigma towards patients with a mental dysregulation for ambulance and emergency department healthcare professionals

Journal:	*BMJ Open*
Manuscript ID	bmjopen-2023-072604.R1
Article Type:	Protocol
Date Submitted by the Author:	06-Sep-2023

Complete List of Authors:	Van de Glind, Geurt; HU University of Applied Sciences Utrecht, Institute of Nursing Studies<br>Galenkamp, Niek; HU University of Applied Sciences Utrecht<br>Bleijenberg, Nienke; HU University of Applied Sciences Utrecht<br>Schoonhoven, Lisette; University Medical Center Utrecht; University of Southampton<br>Scheepers, Floortje E; University Medical Centre Utrecht Brain Centre<br>Crilly, Julia; Griffith University, School of Nursing and Midwifery; Griffith University, Menzies Health Institute Queensland<br>van Veen, Mark; HU University of Applied Sciences Utrecht, Institute of Nursing Studies<br>Ham, Wietske H.W.; HU University of Applied Sciences Utrecht

**Primary Subject Heading**:	Emergency medicine
Secondary Subject Heading:	Mental health
Keywords:	PSYCHIATRY, ACCIDENT & EMERGENCY MEDICINE, MENTAL HEALTH

SCHOLARONE™
Manuscripts

Title

Review protocol for an integrative review on interventions to reduce interpersonal stigma towards patients with a mental dysregulation for ambulance and emergency department healthcare professionals

Corresponding author: Geurt van de Glind

E-mail address of the corresponding author: geurt.vandeglind@hu.nl

Physical mailing address of the corresponding author:

University of Applied Sciences Utrecht

PO box 12011

3501 AA Utrecht

The Netherlands

Authors

Geurt Van de Glind , Niek Galenkamp , Nienke Bleijenberg , Lisette Schoonhoven[2,3], Floortje E. Scheepers[4], Julia Crilly[5, 6], Mark van Veen , Wietske H.W. Ham

University of Applied Sciences, Utrecht, the Netherlands

[2] Nursing Science, Julius Center for Health Sciences and Primary Care, University Medical Center Utrecht/ Utrecht University, Utrecht, The Netherlands;

[3] School of Health Sciences, Faculty of Environmental and Life Sciences, University of Southampton, UK.

[4] University Medical Center Utrecht, The Netherlands

[5] Department of Emergency Medicine, Gold Coast Health, Gold Coast, Queensland, Australia

[6] School of Nursing and Midwifery/ Menzies Health Institute Queensland/ Centre for Mental Health, Griffith University, Gold Coast, Queensland, Australia

Key Words

Emergency Care, Social Stigma, Psychiatry

Word count:

Excluding ADDITIONAL INFORMATION: 2417 words

Including ADDITIONAL INFORMATION: 2680 words

For peer review only

ABSTRACT

Introduction: Worldwide, there is a constant increase in the extent and severity of mental illness. An exacerbation of somatic complaints in this group of people can result in recurring ambulance and emergency department care. The care of patients with a mental dysregulation (i.e., experiencing a mental health problem and disproportionate feelings like fear, anger, sadness, or confusion, possibly with associated behaviors) can be complex and challenging in the emergency care context, possibly evoking a wide variety of feelings, ranging from worry or pity to annoyance and frustration in emergency care staff members. These challenges and feelings may lead to stigma towards patients with a mental dysregulation seeking emergency care. Various interventions have been developed impacting attitude and behavior and minimizing stigma held by health care professionals. However, these interventions are not explicitly aimed at the emergency care context nor do these represent perspectives of healthcare professionals working within this context. Therefore, the aim of the proposed review is to gain insight into interventions targeting healthcare professionals, that minimize stigma including beliefs, attitudes, and behavior towards patients with a mental dysregulation within the emergency care context.

Methods and analysis*:* The protocol for a systematic integrative review is presented, using the PRISMA-P recommendations. Study selection and data-extraction will be performed by two independent reviewers. In each step, an expert with lived experience will comment on process and results. Software applications RefWorks-ProQuest, Rayyan and ATLAS.ti will be used to enhance the quality of the review, efficiency of the research work, and transparency of process and results.

Ethics and dissemination: No ethical approval or safety considerations are required for this review. The proposed review will be submitted to a relevant international journal. Results will be presented at relevant medical scientific conferences.

Registration*:* The proposed review is registered at PROSPERO (https://www.crd.york.ac.uk/prospero/) under number CRD42023390664

STRENGTHS AND LIMITATIONS OF THIS STUDY

Strengths

- To capture as many interventions as possible, in different stages of development, a broad search strategy including a range of considered terms, types of research (qualitative and quantitative) and in ambulance and emergency department setting with no limits to the publication-date, is chosen;

For peer review only

- The quality of included papers will be assessed by two independent reviewers, using an appropriate instrument, to inform readers on the evidence-based strength or weakness of included papers;
- A person with lived experience is part of the review process and will co-author the review.

Limitations

- Publications in English and Dutch only are included and important publications in other languages are therefore not included.

For peer review only

INTRODUCTION

Worldwide, there is a constant increase in the extent and severity of mental illness (1,2), exacerbated by the COVID-19 pandemic (3,4). More than a quarter (5) of the world's population experience a severe mental health problem at some point during their life. The burden of mental illness both on a socio-economic level and on the quality of life is immense (1,2,5). The World Health Organization (WHO) indicates that patients with chronic mental illness are 40-60% more likely to die 10 to 30 years earlier compared to people without a mental illness (5).

People with mental illnesses have an increased risk for other somatic chronic illnesses (6-8). Furthermore, for these people maintaining a healthy lifestyle, financial stability and access to appropriate somatic care are often problematic (9). Apart from (acute situations due to) their mental problems, an exacerbation of somatic complaints in this group of people can result in recurring ambulance and emergency department care (6,8,10-12). We use the term *Emergency Care Context (ECC)* to reflect both Ambulance care and Accident and Emergency department care.

To describe patients with mental health problems who seek emergency care, several definitions or terms are used in the scientific literature. These include patients with 'mental illness' (13), 'mental disorder' (14) or 'mental health crisis' (15). Other authors focus on a specific sub-group of patients with mental health problems who need emergency care, such as patients with 'borderline personality disorders' (16), 'substance use disorders' (17) or 'suicide attempts' (18). The problem with these descriptions is that uniformity is lacking. Moreover, some healthcare conditions (such as mental health disorders) are in many cases initially unknown and uncharted in the ECC. This adds to the reasons why ECC professionals typically focus on vital signs and behaviors in order to chart and treat acute physical healthcare problems. Therefore, the authors of this review protocol propose the concept 'mental dysregulation' for use in researching care for this vulnerable group in the ECC. The authors propose the following definition: '*In the ECC, patients with mental health problems who seek*

emergency care may experience disproportionate feelings like fear, anger, sadness, or confusion, possibly with associated behaviors. When the aforementioned symptoms interfere with the patient's treatment or that of others, the patient is considered to have a mental dysregulation'. This concept and definition will be subject to a Delphi-procedure including international experts on this topic in 2023-2024 for further harmonizing concepts used in international research on this matter.

The care for patients with a mental dysregulation who seek emergency care can be complex and intensive. In addition to a physical disorder or illness, these patients may be confused or emotionally unstable (8,9,19-23), which may hinder the identification and treatment of both physical and mental health symptoms. The provision of care for patients with a mental dysregulation can evoke a wide variety of feelings in ECC staff, ranging from worry or pity, to annoyance and frustration (8,24-27). These feelings may originate from several challenges in the ECC for these patients. First, these challenges may be induced by the still existing and experienced segregation between somatic and mental treatment, long waiting times (28), and revisits of patients with a mental dysregulation which cause irritation and a decrease in confidence in the effectiveness of interventions. Second, healthcare professionals in the ECC do not always have the right knowledge of psychopathology and legislation (21,29) or the skills to use de-escalation techniques in a timely fashion (30,31), which can lead to frustration.

These challenges and feelings may lead to stigma towards patients with a mental dysregulation who seek emergency care (26-29,31,32). Several qualitative studies have revealed that patients with mental problems often feel stigmatized by healthcare professionals in the ECC(15,32-34). A recent scoping review of factors that influence emergency department nurses care for patients with mental illness revealed that the emergency department environment greatly impacts the role of emergency department nurses, that their beliefs and perceptions leads to role conflicts, and the lack of knowledge and confidence impacts their view towards patients with mental illness. Specifically, some nurses reported they avoided patients, judged them as less important and attention seeking (25), which implies stigma towards these group of patients.

In their landmark paper Link & Phelan define stigma as existing when elements of labeling, stereotyping, separating ("us" from "them"), status loss, and discrimination co-occur in a power situation that allows these processes to unfold (35). Thornicroft and colleagues use this definition and elaborate on four different types of stigma of which, for this review, interpersonal stigma (also referred to as 'public stigma') is important. Interpersonal stigma refers to the link between stereotypes, negative attitudes (prejudice), and negative behavior (discrimination) towards people with mental health conditions. Three related components are part of interpersonal stigma:

For peer review only - http://bmjopen.bmj.com/site/about/guidelines.xhtml

knowledge (ignorance or misinformation), attitudes (negative emotional reactions, such as prejudice) and behavior (such as avoidance or rejection) (36).

Various interventions have been developed that impact attitude and behavior and minimize stigma held by health care professionals. Thornicroft and colleagues report on 56 reviews, documenting changes in knowledge, attitudes, and clinical skills as well as clinical confidence and self-efficacy as stigma related outcomes. Several types of interventions are mentioned, such as e-interventions, use of simulations (e.g., standardized role-plays with actors), and social contact, such as filmed or live recovery testimonials. Studies involving different methods of contact (e.g., live or filmed) were more often associated with better outcomes for knowledge and attitudes when compared to educational interventions alone. Interventions including experts with lived experience in the design and evaluation of stigma interventions demonstrated better outcomes (36). The interventions outlined from the reviews covered various populations (e.g., mental health professionals, health care providers) and various mental health problems (e.g., dementia care, suicide prevention, borderline personality disorder); only one publication was specific to emergency department healthcare staff and focused on their attitudes towards patients with substance-related presentations (37). To our knowledge, a comprehensive review of the available scientific evidence in this specific ECC is lacking.

AIM

To review the body of evidence to gain insight into interventions targeting healthcare professionals, that minimize stigma including beliefs, attitudes, and behavior towards patients with a mental dysregulation within the ECC.

CONCEPTS

For this review, we define *patients with a mental dysregulation* in the ECC as follows: patients with mental problems that seek emergency care may experience disproportionate feelings like fear, anger, sadness, or confusion, possibly with associated behaviors. When the aforementioned symptoms interfere with the patient's treatment or that of others, the patient is considered to have a mental dysregulation.

We applied the World Health Organization definition of '*Intervention*', namely, 'an act performed for, with or on behalf of a person or population whose purpose is to assess, improve, maintain, promote, or modify health, functioning or health conditions' (38).

Stigma is defined as the link between stereotypes, negative attitudes, and discrimination against people with mental health conditions in society (36).

For peer review only

Interpersonal stigma encompasses the association between stereotypes, adverse attitudes (prejudice), and detrimental actions (discrimination) directed at individuals dealing with mental health conditions. It comprises three interconnected elements: knowledge (comprising ignorance or misconceptions), attitudes (comprising unfavorable emotional responses, like prejudice), and behavior (encompassing actions such as avoidance or rejection) (36).

The emergency care context (ECC) in this review is defined as emergency care from the scene of acute incident of illness, followed by ambulance transport to and treatment in the emergency department.

Healthcare professionals include paramedics, emergency care nurses, nurse specialists, physicians and physician assistants that are directly involved in providing emergency care.

RESEARCH QUESTIONS

1. Which interventions that address interpersonal stigma towards patients with a mental dysregulation have been designed, implemented and evaluated for healthcare professionals in the ECC?
2. From the healthcare professionals' perspective, which facilitators and barriers were encountered when implementing these interventions within the ECC?
3. From the service users' perspective, which facilitators and barriers were encountered when implementing these interventions within the ECC?

For peer review only

METHODS AND ANALYSIS

We will perform an integrative review of empirical literature using methodological strategies proposed by Whittemore and Knafl(39), using a 7-step framework outlined by Dhollande and colleagues(40) to identify current state of the art knowledge on the topic and to identify current knowledge gaps in research. The 7-steps applied to the proposed integrative review will involve: 1) write the review question; 2) determine the search strategy; 3) critically appraise the search results; 4) summarize the search results; 5) extract and reduce data; 6) analysis; and 7) conclusions and implications (40). An integrative review allows for inclusion of diverse study methodologies, including qualitative, quantitative, experimental, and mixed-method studies, which makes it suitable for topics that have not been studied intensively. Results from an integrative review provide a complete and varied perspective of the studied topic.

To further enhance the quality of the review, the PRISMA-P recommendations (41) will be followed where applicable(41). The PRISMA-P checklist is attached as Appendix I. Software applications RefWorks-ProQuest, Rayyan and ATLAS.ti will be used to enhance the quality of the review, efficiency of the research work, and transparency of process and results.

Patient and Public Involvement

As Thornicroft and colleagues highlight in their publication on stigma it is important to involve people with lived experience of mental health conditions (PWLE) in both research and innovation projects (36). For that reason, one or two PWLE will be included in the project team and co-author the review. For the development of this review protocol no patient or PWLE was involved.

Data sources and search strategy

A systematic search will be applied, followed by identifying possible missed publications via the reference lists of included publications. The following scientific databases will be systematically searched for English and Dutch publications: CINAHL, PsychINFO and MEDLINE. Search terms will include ‘Stigma’, ‘Interpersonal stigma’, ‘Public stigma’, ‘Interventions’, ‘Emergency healthcare’, ‘Emergency healthcare staff’, 'mental health', emergency department', 'ambulance', 'paramedics' and related search terms, as used in the aforementioned databases. The full search strategy is attached as Appendix II. The results will be presented in the review. Additionally, the grey literature will be searched. This grey literature will be limited to National Guidelines related to this topic (ECC) in English or Dutch. We will search via Google and via Guideline Organizations (e.g., The National Institute for Health and Care Excellence, NICE, in the United Kingdom) and professional organizations (e.g., the American Academy of Emergency Medicine).

For peer review only

Study selection and sorting

Studies will be included using the following inclusion and exclusion criteria:

Inclusion

- Original research report, both qualitative and quantitative studies;
- RCTs, prospective and retrospective cohort studies, case control studies, cross sectional studies and, if available, literature reviews;
- Description of innovation projects related to the topic; depending on information on outcome variables (see *Data extraction, analysis, and synthesis*-paragraph below);
- Focused on intervention(s), targeting healthcare professionals, to reduce stigma on mental health problems held by ECC staff;
- Peer reviewed publication;
- Published in English or Dutch.
- To obtain as much information as possible on relevant interventions, the search includes thesis and conference abstracts.

Exclusion

- Abstract and/ or full text not available

Study-selection: The results of the search will be assessed in the following steps: 1) Review of title and abstract and 2) Full text review of selected publications. Publications that clearly do not answer the research question or address the inclusion criteria will be excluded (i.e., other topic, not an ECC, no stigma related intervention, language). Both steps will be performed by two independent reviewers, using Rayyan, a web-based tool, designed for publications-selection in scientific literature review (42). When no consensus is reached on a particular publication a third reviewer will make a final decision. A PRISMA flow diagram will be used to provide a summary of the search and selection process.

Quality appraisal

The quality of included studies will be assessed with the Mixed Methods Appraisal Tool (43), developed and improved by Hong and colleagues (44). Each included publication will be assessed by two independent reviewers. Quality appraisal is not used for study selection, but for means of interpreting the strength of findings and conclusions. As Thornicrofft and colleagues (36) emphasize the importance of including persons with lived experience in the development of interventions, we

For peer review only

will include this in reviewing and appraising interventions presented in the included publications, by answering this question: To what extent are people with lived experience involved in the design/ delivery/ evaluation of the intervention and if involved, what were the results of this?

Data extraction, analysis, and synthesis

Data extraction will be performed using Atlas.ti, a software application, originally designed for qualitative research analyses purposes, but also used for transparent data extraction and analyses in literature review (45). A predetermined set of labels will be set up within ATLAS.ti and will include (but is not limited to): definition of stigma, description of intervention, study population, study design, sample, site/setting, measurements, outcome variable(s), reported strengths of the study, reported limitations of the study.

The main outcome variables of interest are 1) Level of interpersonal stigma and 2) patient satisfaction. The secondary outcome variable of interest is: Level of knowledge on psychiatry.

Depending on the instruments used in the original publications, the measures and instruments for results on the outcome variables will be listed, when appropriate in table(s). In the discussion part of the review, the quality of the used instruments will be discussed.

Two independent reviewers will analyze the included publications, identifying (by adding the predefined labels to) citations in the publications, relevant for answering the review questions. Depending on the content of the results, we will, when appropriate, perform thematic analysis as described by Dhollande and colleagues(40).

The selected citations will be further analyzed and discussed by the authors, using ATLAS.ti tools, whereafter the extracted data will be used for answering the research questions.

Ethics and dissemination

No ethical approval or safety considerations are required for this review. The proposed review will be submitted to a relevant peer reviewed international journal. Results will be presented at relevant medical scientific conferences.

For peer review only

ADDITIONAL INFORMATION

Planning and time-frame

Start search: Immediately after first submission of the review protocol: February 2023 and repeated in July 2023.

Study selection: February/August 2023

Data extraction and synthesis: April/September 2023

Writing and submission of the review: October/November 2023

Author Statement

GVdG, NG, WHWH developed the initial protocol: review questions, introduction and methods. NB, LS, FS, JC and MvV commented on the manuscript and re-wrote sections of the manuscript. All authors documented their approval of the final version of the review protocol before submission. As senior researcher and project-leader WHWH is the guarantor of the review and review protocol.

Data Statement

As ATLAS.ti is used for data extraction, analysis and synthesis, used citations including used labels from included publications are available via the corresponding author.

Declaration of Conflicting Interests

The authors declare to have no conflicts of interest related to this review.

Funding statement

This review is part of the PONTAC-study (Psychisch ONTregeld in de Acute zorg/ Mental dysregulation in Acute healthcare). The PONTAC study is co-financed by SIA-RAAK, part of the Netherlands Organization for Scientific Research (NWO), under award/grant number: RAAK.PUB08.029. Furthermore, this study was partly funded by a fellowship funding from the Netherlands Organisation for Health Research and Development (ZonMw), under award/grant number number 10040022110004, was awarded to dr. Wietske H.W. Ham (Wietske Blom-Ham).

The University of Applied Sciences, Utrecht, University Medical Centre, Utrecht, Diakonessenhuis, Utrecht, St.Antionius Hospital, Utrecht, Meander Medical Center, Amersfoort, Regional Ambulance

For peer review only

Service Utrecht, Altrecht, Mental Healthcare, Utrecht have contributed in-kind to the study. The latter 7 institutions did not provide an award/grant number to this project.

For peer review only

References

(1) Whiteford HA, Ferrari AJ, Degenhardt L, Feigin V, Vos T. The global burden of mental, neurological and substance use disorders: an analysis from the Global Burden of Disease Study 2010. PLoS One 2015;10(2):e0116820.

(2) Arias D, Saxena S, Verguet S. Quantifying the global burden of mental disorders and their economic value. EClinicalMedicine 2022 Sep 28;54:101675.

(3) Boden M, Zimmerman L, Azevedo KJ, Ruzek JI, Gala S, Abdel Magid H,S., et al. Addressing the mental health impact of COVID-19 through population health; PMC7934657. Clin.Psychol.Rev. 2021;85(102006):102006.

(4) Xiong J, Lipsitz O, Nasri F, Lui LMW, Gill H, Phan L, et al. Impact of COVID-19 pandemic on mental health in the general population: A systematic review; PMC7413844. J.Affect.Disord. 2020;277:55-64.

(5) Kessler RC, Aguilar-Gaxiola S, Alonso J, Chatterji S, Lee S, Ormel J, et al. The global burden of mental disorders: an update from the WHO World Mental Health (WMH) surveys; PMC3039289. Epidemiol.Psichiatr.Soc. 2009;18(1):23-33.

(6) Scott KM, Lim C, Al-Hamzawi A, Alonso J, Bruffaerts R, Caldas-de-Almeida JM, et al. Association of mental disorders with subsequent chronic physical conditions: World mental health surveys from 17 countries; PMC5333921. JAMA Psychiatry 2016;73(2):150-158.

(7) Momen NC, Plana-Ripoll O, Agerbo E, Benros ME, Børglum A,D., Christensen MK, et al. Association between mental disorders and subsequent medical conditions; PMC7261506. N.Engl.J.Med. 2020;382(18):1721-1731.

(8) Giandinoto J, Edward K. Challenges in acute care of people with co-morbid mental illness. Br J Nurs 2014 Jul 10-23;23(13):728-732.

(9) Giandinoto J, Edward K. The phenomenon of co-morbid physical and mental illness in acute medical care: the lived experience of Australian health professionals. BMC Res Notes 2015 Jul 07,;8:295.

(10) Theriault KM, Rosenheck RA, Rhee TG. Increasing Emergency Department Visits for Mental Health Conditions in the United States. J Clin Psychiatry 2020 Jul 28,;81(5).

(11) Ronaldson A, Elton L, Jayakumar S, Jieman A, Halvorsrud K, Bhui K. Severe mental illness and health service utilisation for nonpsychiatric medical disorders: A systematic review and meta-analysis. PLoS Med 2020 09;17(9):e1003284.

(12) Liu NH, Daumit GL, Dua T, Aquila R, Charlson F, Cuijpers P, et al. Excess mortality in persons with severe mental disorders: a multilevel intervention framework and priorities for clinical practice, policy and research agendas; PMC5269481. World Psychiatry 2017;16(1):30-40.

(13) Azar KMJ, Petersen JP, Shen Z, Nasrallah C, Pesa J, LaMori J, et al. Serious Mental Illness and Health-Related Factors Associated with Regional Emergency Department Utilization. Popul Health Manag 2020 Dec;23(6):430-437.

For peer review only

(14) Armoon B, Cao Z, Grenier G, Meng X, Fleury M. Profiles of high emergency department users with mental disorders. Am J Emerg Med 2022;54:131-141.

(15) Sacre M, Albert R, Hoe J. What are the experiences and the perceptions of service users attending Emergency Department for a mental health crisis? A systematic review. Int.J.Ment.Health Nurs. 2022;31(2):400-423.

(16) Artioli M, Bougon E, Mathur A, Salles J. Factors associated with a borderline personality disorder diagnosis in the emergency department. Front Psychiatry 2022;13:925462.

(17) Dowdell EB, Alderman SE, Foushee N, Holland E, Reedy EA. Expressions of Compassion Fatigue by Emergency Department Nurses Caring for Patients With Opioid and Substance Use Disorders. J Emerg Nurs 2022;48(6):688-697.

(18) Rassy J, Daneau D, Larue C, Rahme E, Low N, Lamarre S, et al. Measuring Quality of Care Received by Suicide Attempters in the Emergency Department. Arch Suicide Res 2022;26(1):81-90.

(19) Innes K, Morphet J, O'Brien AP, Munro I. Caring for the mental illness patient in emergency departments--an exploration of the issues from a healthcare provider perspective. J Clin Nurs 2014 Jul;23(13-14):2003-2011.

(20) van Nieuwenhuizen A, Henderson C, Kassam A, Graham T, Murray J, Howard LM, et al. Emergency department staff views and experiences on diagnostic overshadowing related to people with mental illness. Epidemiol Psychiatr Sci 2013 Sep;22(3):255-262.

(21) Marynowski-Traczyk D, Moxham L, Broadbent M. Emergency Department Registered Nurses' conceptualisation of recovery for people experiencing mental illness. Australas Emerg Nurs J 2017 May;20(2):75-81.

(22) Rolfe U, Pope C, Crouch R. Paramedic performance when managing patients experiencing mental health issues - Exploring paramedics' Presentation of Self. Int Emerg Nurs 2020 03;49:100828.

(23) Shefer G, Henderson C, Howard LM, Murray J, Thornicroft G. Diagnostic overshadowing and other challenges involved in the diagnostic process of patients with mental illness who present in emergency departments with physical symptoms--a qualitative study. PLoS One 2014;9(11):e111682.

(24) Beks H, Healey C, Schlicht KG. 'When you're it': a qualitative study exploring the rural nurse experience of managing acute mental health presentations. Rural Remote Health 2018 08;18(3):4616.

(25) Perrone McIntosh J,T. Emergency department nurses' care of psychiatric patients: A scoping review. Int.Emerg.Nurs. 2021;54(100929):100929.

(26) Clarke D, Usick R, Sanderson A, Giles-Smith L, Baker J. Emergency department staff attitudes towards mental health consumers: a literature review and thematic content analysis. Int J Ment Health Nurs 2014 Jun;23(3):273-284.

(27) Emond K, O'Meara P, Bish M. Paramedic management of mental health related presentations: a scoping review. J Ment Health 2019 Feb;28(1):89-96.

For peer review only

(28) Sweeny A, Keijzers G, O'Dwyer J, Stapelberg NC, Crilly J. Patients with mental health conditions in the emergency department: Why so long a wait? Emerg Med Australas 2020 Jun 08,.

(29) Mérelle SYM, Boerema I, van der Linden MC, Gilissen R. [Issues in emergency care for people who attempted suicide]. Ned Tijdschr Geneeskd 2018 Jul 02,;162.

(30) Rolfe U, Pope C, Crouch R. Paramedic performance when managing patients experiencing mental health issues - Exploring paramedics' Presentation of Self. Int Emerg Nurs 2020 03;49:100828.

(31) McCann TV, Savic M, Ferguson N, Cheetham A, Witt K, Emond K, et al. Recognition of, and attitudes towards, people with depression and psychosis with/without alcohol and other drug problems: results from a national survey of Australian paramedics. BMJ Open 2018 12 04,;8(12):e023860.

(32) Wong AH, Ray JM, Rosenberg A, Crispino L, Parker J, McVaney C, et al. Experiences of Individuals Who Were Physically Restrained in the Emergency Department. JAMA Netw Open 2020 01 03,;3(1):e1919381.

(33) Daggenvoorde TH, Gijsman HJ, Goossens PJJ. Emergency care in case of acute psychotic and/or manic symptoms: Lived experiences of patients and their families with the first interventions of a mobile crisis team. A phenomenological study. Perspect Psychiatr Care 2018 Oct;54(4):462-468.

(34) Digel Vandyk A, Young L, MacPhee C, Gillis K. Exploring the Experiences of Persons Who Frequently Visit the Emergency Department for Mental Health-Related Reasons. Qual Health Res 2018 03;28(4):587-599.

(35) Link, Bruce G. Phelan, Jo C. Conceptualizing Stigma. . annurev.soc. 2001;27:363-385.

(36) Thornicroft G, Sunkel C, Alikhon Aliev A, Baker S, Brohan E, El Chammay R, et al. The Lancet Commission on ending stigma and discrimination in mental health. Lancet 2022;400(10361):1438-1480.

(37) Gonzalez M, Clarke DE, Pereira A, Boyce-Gaudreau K, Waldman C, Demczuk L, et al. The impact of educational interventions on attitudes of emergency department staff towards patients with substance-related presentations: a quantitative systematic review. JBI Database System Rev Implement Rep 2017 Aug;15(8):2153-2181.

(38) Fortune N, Madden R, Almborg A. Use of a new international classification of Health Interventions for capturing information on Health Interventions relevant to people with disabilities; PMC5800244. Int.J.Environ.Res.Public Health 2018;15(1).

(39) Whittemore R, Knafl K. The integrative review: updated methodology. J.Adv.Nurs. 2005;52(5):546-553.

(40) Dhollande S, Taylor A, Meyer S, Scott M. Conducting integrative reviews: a guide for novice nursing researchers. J Res Nurs 2021 Aug;26(5):427-438.

(41) Shamseer L, Moher D, Clarke M, Ghersi D, Liberati A, Petticrew M, et al. Preferred reporting items for systematic review and meta-analysis protocols (PRISMA-P) 2015: elaboration and explanation. BMJ 2015 Jan 2;350:g7647.

For peer review only

(42) Rayyan. Faster systematic reviews. 2023; Available at: https://www.rayyan.ai/. Accessed 03-21-, 2023.

(43) Mixed Methods Appraisal Tool - version 2018. 2018; Available at: http://mixedmethodsappraisaltoolpublic.pbworks.com/w/file/fetch/127916259/MMAT_2018_criteria-manual_2018-08-01_ENG.pdf. Accessed 20th December, 2022.

(44) Hong QN, Gonzalez-Reyes A, Pluye P. Improving the usefulness of a tool for appraising the quality of qualitative, quantitative and mixed methods studies, the Mixed Methods Appraisal Tool (MMAT). J.Eval.Clin.Pract. 2018;24(3):459-467.

(45) Smit B, Scherman V. Computer-Assisted Qualitative Data Analysis Software for Scoping Reviews: A Case of ATLAS.ti. International Journal of Qualitative Methods 2021;20:16094069211019140.

For peer review only

Appendix I: PRISMA-P checklist

Section and topic	Item No	Checklist item	How and where is the item addressed in this review protocol
**ADMINISTRATIVE INFORMATION**			
Title:			
Identification	1a	Identify the report as a protocol of a systematic review	the title and methods describe the review as an integrative review.
Update	1b	If the protocol is for an update of a previous systematic review, identify as such	Not applicable
Registration	2	If registered, provide the name of the registry (such as PROSPERO) and registration number	PROSPERO ID number: CRD42023390664
Authors:			
Contact	3a	Provide name, institutional affiliation, e-mail address of all protocol authors; provide physical mailing address of corresponding author	Name, affiliations of all protocol authors are listed. Email address and physical mailing address of the corresponding author is provided on the title page. E-mail addresses of co-authors are presented to the publisher of the review protocol.
Contributions	3b	Describe contributions of protocol authors and identify the guarantor of the review	GVdG, NG, WHWH developed the initial protocol: review questions, introduction and methods. NB, LS, FS, JC, MvV commented on the manuscript and re-wrote sections of the manuscript. All authors documented their approval of the final version of the review protocol before submission. As senior researcher and project-leader WHWH is the guarantor of the review and review protocol.
Amendments	4	If the protocol represents an amendment of a previously completed or published protocol, identify as such and list changes; otherwise, state plan for documenting important protocol amendments	n.a.
Support:			
Sources	5a	Indicate sources of financial or other support for the review	Provided in the section 'Funding Statement'
Sponsor	5b	Provide name for the review funder and/or sponsor	Provided in the section 'Funding Statement'
Role of sponsor or funder	5c	Describe roles of funder(s), sponsor(s), and/or institution(s), if any, in developing the protocol	The funders/institutions have no role in developing the protocol. The authors, though working for some of these institutions, work independently on this research.
**INTRODUCTION**			
Rationale	6	Describe the rationale for the review in the context of what is already known	Provided in the introduction
Objectives	7	Provide an explicit statement of the question(s) the review will address with reference to participants, interventions, comparators, and outcomes (PICO)	Participants/Interventions: Provided in the Concepts/Aim/Research questions/ Methods

For peer review only

			Comparators will be documented, dependent on what is given in original publications.<br>Outcome variables: Main outcome variables: 1)Level of Interpersonal Stigma and 2) Patient satisfaction. The secondary outcome variable looked at is: Level of knowledge on psychiatry.
**METHODS**			
Eligibility criteria	8	Specify the study characteristics (such as PICO, study design, setting, time frame) and report characteristics (such as years considered, language, publication status) to be used as criteria for eligibility for the review	All addressed in the protocol, in the 'Study selection and sorting'- section.
Information sources	9	Describe all intended information sources (such as electronic databases, contact with study authors, trial registers or other grey literature sources) with planned dates of coverage	Listed in the Methods section: Medline, PsychInfo, CINAHL.<br>Grey literature: Search via Google and contacting Professional Organizations for National Guidelines on the topic, in English and Dutch speaking countries.
Search strategy	10	Present draft of search strategy to be used for at least one electronic database, including planned limits, such that it could be repeated	The full search strategy is attached as Appendix II to this review protocol.
Study records:			
Data management	11a	Describe the mechanism(s) that will be used to manage records and data throughout the review	Refworks Proquest will be used for management of the records.<br>Rayyan will be used for independent selection of studies, by two independent reviewers. Atlas.ti.23 will be used for data-extraction.
Selection process	11b	State the process that will be used for selecting studies (such as two independent reviewers) through each phase of the review (that is, screening, eligibility and inclusion in meta-analysis)	Addressed in the 'Study selection and sorting' section. All steps in screening, in- and exclusion process, data-extraction and appraisal is carried out by two independent reviewers.
Data collection process	11c	Describe planned method of extracting data from reports (such as piloting forms, done independently, in duplicate), any processes for obtaining and confirming data from investigators	Addressed in the 'Data extraction, analysis, and synthesis' section. Data extraction to be performed by two independent reviewers. Using ATLAS.ti.
Data items	12	List and define all variables for which data will be sought (such as PICO items, funding sources), any pre-planned data assumptions and simplifications	Addressed in the 'Concepts' section.
Outcomes and prioritization	13	List and define all outcomes for which data will be sought, including prioritization of main and additional outcomes, with rationale	Provided in the 'Data extraction, analysis, and synthesis' section.
Risk of bias in individual studies	14	Describe anticipated methods for assessing risk of bias of individual studies, including whether this will be done at the outcome	Addressed in the 'Quality appraisal' section. Each included publication will be rated using the MMAT by two independent reviewers.

For peer review only

		or study level, or both; state how this information will be used in data synthesis	
Data synthesis	15a	Describe criteria under which study data will be quantitatively synthesized	a. and b. Given the nature of the outcome variables, and the expected paucity in available original research, we expect not to be able to quantitatively synthesize results.
	15b	If data are appropriate for quantitative synthesis, describe planned summary measures, methods of handling data and methods of combining data from studies, including any planned exploration of consistency (such as I^2, Kendall's τ)	Included studies will be listed in tables, presenting the main characteristics of the included studies: type of innovation, level of development, outcome measures, type of evaluation/testing of the innovations.
	15c	Describe any proposed additional analyses (such as sensitivity or subgroup analyses, meta-regression)	For each of the included studies, the results of the quality appraisal, using the MMAT (Mixed Methods Appraisal Tool) will be documented.
	15d	If quantitative synthesis is not appropriate, describe the type of summary planned	
Meta-bias(es)	16	Specify any planned assessment of meta-bias(es) (such as publication bias across studies, selective reporting within studies)	Given the expected paucity in available original research we do not expect Meta-bias(es).
Confidence in cumulative evidence	17	Describe how the strength of the body of evidence will be assessed (such as GRADE)	We will use the MMAT results as an indication for the overall quality of included publications. If the number and quality of included publications exceeds expectations, the authors will decide on using GRADE as an instrument for grading the quality of the included research.

For peer review only

Appendix II: Search Strategy

Medline/Pubmed:

Search	Query
#1	Attitude of Health Personnel[Mesh] OR Social Stigma[Mesh] OR Professional Competence[Mesh] OR “Professional-Patient Relations”[Mesh] OR “Social Discrimination”[Mesh] OR Attitude*[tiab] OR Stigma[tiab] OR Competence[tiab] OR “Professional Patient Relation*”[tiab] OR Discrimination[tiab] OR Traumatization[tiab] OR Traumatisation[tiab] OR “Secondary Trauma*”[tiab]
#2	Emergency Nursing[Mesh] OR "Emergency Nurs*"[tiab] OR "Emergency Room Nurs*"[tiab] OR "Trauma Nursing"[tiab] OR Emergency Medical Services[Mesh] OR Hospital Emergency Service[Mesh] OR "Emergency Medical Service*"[tiab] OR "Emergency Care"[tiab] OR "Emergency Health Service*"[tiab] OR "Emergency Department*"[tiab] OR "Emergency Room*"[tiab] OR "Emergency Unit*"[tiab] OR "Emergency Ward*"[tiab] OR "Emergency Hospital*"[tiab] OR "Emergency Service*"[tiab] OR Paramedics[Mesh] OR Paramedic*[tiab] OR "Emergency Physician*"[tiab] OR Ambulance*[tiab] OR "First Responder*"[tiab]
#3	Mental Disorders[Mesh] OR "Mental Disorder*"[tiab] OR "Mental Health Issue*"[tiab] OR "Mental Illness*"[tiab] OR "Mental Health Presentation*"[tiab] OR "Mental Health Problem*"[tiab] OR "Psychiatric Disorder*"[tiab] OR "Psychiatric Disease*"[tiab] OR "Psychiatric Illness*"[tiab] OR "Psychiatric Condition*"[tiab] OR Psychophysiologic Disorders[Mesh] OR "Psychophysiologic Disorder*"[tiab] OR "Psychosomatic Disorder*"[tiab] OR Aggression[Mesh] OR Aggression*[tiab] OR Problem Behavior[Mesh] OR "Problem Behavior*"[tiab] OR "Behavioral Problem*"[tiab] OR "Disruptive Behavior*"[tiab] OR "Behavioral Disorder*"[tiab] OR "Psychologically disturbed"[tiab] OR Self-Injurious Behavior[Mesh] OR "Self Injur*"[tiab] OR "Self Harm"[tiab] OR "Self Destruct*"[tiab] OR "Self Mutilat*"[tiab] OR Suicide[tiab] OR Suicidal[tiab]
#4	Intervention*[tiab] OR Action*[tiab] OR Educat*[tiab] OR Knowledge[tiab] OR Consult*[tiab] OR Training[tiab] OR Course*[tiab] OR Treatment[tiab] OR Therap*[tiab] OR Medication[tiab] OR Protocol*[tiab] OR Instrument*[tiab] OR Tool*[tiab]
#5	#1 AND #2 AND #3 AND #4

For peer review only

CINAHL:

Search	Query
#1	MH ("Attitude of Health Personnel+" OR "Professional Competence+" OR Stigma OR "Professional-Patient Relations+" OR Discrimination+) OR TI (Attitude* OR Stigma OR Competence OR "Professional Patient Relation*"OR Discrimination OR Traumatization OR Traumatisation OR "Secondary Trauma*") OR AB (Attitude* OR Stigma OR Competence OR "Professional Patient Relation*" OR Discrimination OR Traumatization OR Traumatisation OR "Secondary Trauma*")
#2	MH ("Emergency Medical Services+" OR "Emergency Nursing+" OR "Emergency Medical Technicians") OR TI ("Emergency Nurs*" OR "Emergency Room Nurs*" OR "Trauma Nursing" OR "Emergency Medical Service*" OR "Emergency Care" OR "Emergency Health Service*" OR "Emergency Department*" OR "Emergency Room*" OR "Emergency Unit*" OR "Emergency Ward*" OR "Emergency Hospital*" OR "Emergency Service*" OR Paramedic* OR "Emergency Physician*" OR Ambulance* OR "First Responder*") OR AB ("Emergency Nurs*" OR "Emergency Room Nurs*" OR "Trauma Nursing" OR "Emergency Medical Service*" OR "Emergency Care" OR "Emergency Health Service*" OR "Emergency Department*" OR "Emergency Room*" OR "Emergency Unit*" OR "Emergency Ward*" OR "Emergency Hospital*" OR "Emergency Service*" OR Paramedic* OR "Emergency Physician*" OR Ambulance* OR "First Responder*")
#3	(MH ("Mental Disorders+" OR "Suicide+" OR "Self-Injurious Behavior") OR TI ("Mental Disorder*" OR "Mental Health Issue*" OR "Mental Illness*" OR "Mental Health Presentation*" OR "Psychiatric Disorder*" OR "Psychiatric Disease*" OR "Psychiatric Illness*" OR "Psychiatric Condition*" OR "Psychophysiologic Disorder*" OR "Psychosomatic Disorder*" OR Aggression* OR Aggressive OR "Problem Behavior*" OR "Behavioral Problem*" OR "Behavior Problem*" OR "Behavior Disorder*" OR "Disruptive Behavior*" OR "Behavioral Disorder*" OR "Psychologically disturbed" OR "Self Injur*" OR "Self Harm" OR "Self Destruct*" OR "Self Mutilat*" OR Suicide OR Suicidal) OR AB ("Mental Disorder*" OR "Mental Health Issue*" OR "Mental Illness*" OR "Mental Health Presentation*" OR "Psychiatric Disorder*" OR "Psychiatric Disease*" OR "Psychiatric Illness*" OR "Psychiatric Condition*" OR "Psychophysiologic Disorder*" OR "Psychosomatic Disorder*" OR Aggression* OR Aggressive OR "Problem Behavior*" OR "Behavioral Problem*" OR "Behavior Problem*" OR "Behavior Disorder*" OR "Psychologically disturbed" OR "Self Injur*" OR "Self Harm" OR "Self Destruct*" OR "Self Mutilat*" OR Suicide OR Suicidal)
#4	TI(Intervention* OR Action* OR Educat* OR Knowledge OR Consult* OR Training OR Course* OR Treatment OR Therap* OR Medication OR Protocol* OR Instrument* OR Tool*) OR

For peer review only

	AB(Intervention* OR Action* OR Educat* OR Knowledge OR Consult* OR Training OR Course* OR Treatment OR Therap* OR Medication OR Protocol* OR Instrument* OR Tool*)
#5	#1 AND #2 AND #3 AND #4

PsycINFO:

Search	Query
#1	DE (Stigma OR "Health Personnel Attitudes" OR "Professional Competence+" OR Discrimination+) OR TI (Attitude* OR Stigma OR Competence OR "Professional Patient Relation*" OR Discrimination OR Traumatization OR Traumatisation OR "Secondary Trauma*") OR AB (Attitude* OR Stigma OR Competence OR "Professional Patient Relation*" OR Discrimination OR Traumatization OR Traumatisation OR "Secondary Trauma*")
#2	DE ("Emergency Services+" OR "Emergency Personnel+") OR TI ("Emergency Nurs*" OR "Emergency Room Nurs*" OR "Trauma Nursing" OR "Emergency Medical Service*" OR "Emergency Care" OR "Emergency Health Service*" OR "Emergency Department*" OR "Emergency Room*" OR "Emergency Unit*" OR "Emergency Ward*" OR "Emergency Hospital*" OR "Emergency Service*" OR Paramedic* OR "Emergency Physician*" OR Ambulance* OR "First Responder*") OR AB ("Emergency Nurs*" OR "Emergency Room Nurs*" OR "Trauma Nursing" OR "Emergency Medical Service*" OR "Emergency Care" OR "Emergency Health Service*" OR "Emergency Department*" OR "Emergency Room*" OR "Emergency Unit*" OR "Emergency Ward*" OR "Emergency Hospital*" OR "Emergency Service*" OR Paramedic* OR "Emergency Physician*" OR Ambulance* OR "First Responder*")
#3	((DE ("Mental Disorders+" OR "Behavior Disorders+" OR "Behavior Problems+" OR "Aggressive Behavior+" OR "Self-Destructive Behavior+") OR TI ("Mental Disorder*" OR "Mental Health Issue*" OR "Mental Illness*" OR "Mental Health Presentation*" OR "Psychiatric Disorder*" OR "Psychiatric Disease*" OR "Psychiatric Illness*" OR "Psychophysiologic Disorder*" OR "Psychosomatic Disorder*" OR Aggression* OR Aggressive OR "Problem Behavior*" OR "Behavioral Problem*" OR "Behavior Problem*" OR "Behavior Disorder*" OR "Disruptive Behavior*" OR "Behavioral Disorder*" OR "Psychologically disturbed" OR "Self Injur*" OR "Self Harm" OR "Self Destruct*" OR "Self Mutilat*" OR Suicide OR Suicidal) OR AB ("Mental Disorder*" OR "Mental Health Issue*" OR "Mental Illness*" OR "Mental Health Presentation*" OR "Psychiatric Disorder*" OR "Psychiatric Disease*" OR "Psychiatric Illness*" OR "Psychophysiologic Disorder*" OR "Psychosomatic Disorder*" OR Aggression* OR Aggressive OR "Problem Behavior*" OR "Behavioral Problem*" OR "Behavior Problem*" OR "Behavior

For peer review only

	Disorder*" OR "Disruptive Behavior*" OR "Behavioral Disorder*" OR "Psychologically disturbed" OR "Self Injur*" OR "Self Harm" OR "Self Destruct*" OR "Self Mutilat*" OR Suicide OR Suicidal))
#4	TI(Intervention* OR Action* OR Educat* OR Knowledge OR Consult* OR Training OR Course* OR Treatment OR Therap* OR Medication OR Protocol* OR Instrument* OR Tool*) OR AB(Intervention* OR Action* OR Educat* OR Knowledge OR Consult* OR Training OR Course* OR Treatment OR Therap* OR Medication OR Protocol* OR Instrument* OR Tool*)
#5	#1 AND #2 AND #3 AND #4

For peer review only

BMJ Open

Review protocol for an integrative review on interventions to reduce interpersonal stigma towards patients with a mental dysregulation for ambulance and emergency department healthcare professionals

Journal:	*BMJ Open*
Manuscript ID	bmjopen-2023-072604.R2
Article Type:	Protocol
Date Submitted by the Author:	28-Sep-2023
Complete List of Authors:	Van de Glind, Geurt; HU University of Applied Sciences Utrecht, Institute of Nursing Studies<br>Galenkamp, Niek; HU University of Applied Sciences Utrecht<br>Bleijenberg, Nienke; HU University of Applied Sciences Utrecht<br>Schoonhoven, Lisette; University Medical Center Utrecht; University of Southampton<br>Scheepers, Floortje E; University Medical Centre Utrecht Brain Centre<br>Crilly, Julia; Griffith University, School of Nursing and Midwifery; Griffith University, Menzies Health Institute Queensland<br>van Veen, Mark; HU University of Applied Sciences Utrecht, Institute of Nursing Studies<br>Ham, Wietske H.W.; HU University of Applied Sciences Utrecht
**Primary Subject Heading**:	Emergency medicine
Secondary Subject Heading:	Mental health
Keywords:	PSYCHIATRY, ACCIDENT & EMERGENCY MEDICINE, MENTAL HEALTH

SCHOLARONE™
Manuscripts

Title

Review protocol for an integrative review on interventions to reduce interpersonal stigma towards patients with a mental dysregulation for ambulance and emergency department healthcare professionals

Corresponding author: Geurt van de Glind

E-mail address of the corresponding author: geurt.vandeglind@hu.nl

Physical mailing address of the corresponding author:

University of Applied Sciences Utrecht

PO box 12011

3501 AA Utrecht

The Netherlands

For peer review only

Authors

Geurt Van de Glind , Niek Galenkamp , Nienke Bleijenberg , Lisette Schoonhoven[2,3], Floortje E. Scheepers[4], Julia Crilly[5, 6], Mark van Veen , Wietske H.W. Ham

University of Applied Sciences, Utrecht, the Netherlands

[2] Nursing Science, Julius Center for Health Sciences and Primary Care, University Medical Center Utrecht/ Utrecht University, Utrecht, The Netherlands;

[3] School of Health Sciences, Faculty of Environmental and Life Sciences, University of Southampton, UK.

[4] University Medical Center Utrecht, The Netherlands

[5] Department of Emergency Medicine, Gold Coast Health, Gold Coast, Queensland, Australia

[6] School of Nursing and Midwifery/ Menzies Health Institute Queensland/ Centre for Mental Health, Griffith University, Gold Coast, Queensland, Australia

Key Words

Emergency Care, Social Stigma, Psychiatry

Word count:

Excluding ADDITIONAL INFORMATION: 2417 words

Including ADDITIONAL INFORMATION: 2680 words

For peer review only - http://bmjopen.bmj.com/site/about/guidelines.xhtml

ABSTRACT

Introduction: Worldwide, there is an increase in the extent and severity of mental illness. An exacerbation of somatic complaints in this group of people can result in recurring ambulance and emergency department care. The care of patients with a mental dysregulation (i.e., experiencing a mental health problem and disproportionate feelings like fear, anger, sadness, or confusion, possibly with associated behaviors) can be complex and challenging in the emergency care context, possibly evoking a wide variety of feelings, ranging from worry or pity to annoyance and frustration in emergency care staff members. This in return may lead to stigma towards patients with a mental dysregulation seeking emergency care. Interventions have been developed impacting attitude and behavior and minimizing stigma held by health care professionals. However, these interventions are not explicitly aimed at the emergency care context nor do these represent perspectives of healthcare professionals working within this context. Therefore, the aim of the proposed review is to gain insight into interventions targeting healthcare professionals, that minimize stigma including beliefs, attitudes, and behavior towards patients with a mental dysregulation within the emergency care context.

Methods and analysis*:* The protocol for a systematic integrative review is presented, using the PRISMA-P recommendations. A systematic search was performed 13th July 2023. Study selection and data-extraction will be performed by two independent reviewers. In each step, an expert with lived experience will comment on process and results. Software applications RefWorks-ProQuest, Rayyan and ATLAS.ti will be used to enhance the quality of the review and transparency of process and results.

Ethics and dissemination: No ethical approval or safety considerations are required for this review. The proposed review will be submitted to a relevant international journal. Results will be presented at relevant medical scientific conferences.

Registration*:* The proposed review is registered at PROSPERO (https://www.crd.york.ac.uk/prospero/) under number CRD42023390664

STRENGTHS AND LIMITATIONS OF THIS STUDY

Strengths

- To capture as many interventions as possible, in different stages of development, a broad search strategy including a range of considered terms, types of research (qualitative and quantitative)

For peer review only - http://bmjopen.bmj.com/site/about/guidelines.xhtml

and in ambulance and emergency department setting with no limits to the publication-date, is chosen;

- The quality of included papers will be assessed by two independent reviewers, using an appropriate instrument, to inform readers on the evidence-based strength or weakness of included papers;
- A person with lived experience is part of the review process and will co-author the review.

Limitations

- Publications in English and Dutch only are included and important publications in other languages are therefore not included.

INTRODUCTION

Worldwide, there is a constant increase in the extent and severity of mental illness (1,2), exacerbated by the COVID-19 pandemic (3,4). More than a quarter (5) of the world's population experience a severe mental health problem at some point during their life. The burden of mental illness both on a socio-economic level and on the quality of life is immense (1,2,5). The World Health Organization (WHO) indicates that patients with chronic mental illness are 40-60% more likely to die 10 to 30 years earlier compared to people without a mental illness (5).

People with mental illnesses have an increased risk for other somatic chronic illnesses (6-8). Furthermore, for these people maintaining a healthy lifestyle, financial stability and access to appropriate somatic care are often problematic (9). Apart from (acute situations due to) their mental problems, an exacerbation of somatic complaints in this group of people can result in recurring ambulance and emergency department care (6,8,10-12). We use the term *Emergency Care Context (ECC)* to reflect both Ambulance care and Accident and Emergency department care.

To describe patients with mental health problems who seek emergency care, several definitions or terms are used in the scientific literature. These include patients with 'mental illness' (13), 'mental disorder' (14) or 'mental health crisis' (15). Other authors focus on a specific sub-group of patients with mental health problems who need emergency care, such as patients with 'borderline personality disorders' (16), 'substance use disorders' (17) or 'suicide attempts' (18). The problem with these descriptions is that uniformity is lacking. Moreover, some healthcare conditions (such as mental health disorders) are in many cases initially unknown and uncharted in the ECC. This adds to the reasons why ECC professionals typically focus on vital signs and behaviors in order to chart and

For peer review only - http://bmjopen.bmj.com/site/about/guidelines.xhtml

treat acute physical healthcare problems. Therefore, the authors of this review protocol propose the concept 'mental dysregulation' for use in researching care for this vulnerable group in the ECC. The authors propose the following definition: '*In the ECC, patients with mental health problems who seek emergency care may experience disproportionate feelings like fear, anger, sadness, or confusion, possibly with associated behaviors. When the aforementioned symptoms interfere with the patient's treatment or that of others, the patient is considered to have a mental dysregulation'*. This concept and definition will be subject to a Delphi-procedure including international experts on this topic in 2023-2024 for further harmonizing concepts used in international research on this matter.

The care for patients with a mental dysregulation who seek emergency care can be complex and intensive. In addition to a physical disorder or illness, these patients may be confused or emotionally unstable (8,9,19-23), which may hinder the identification and treatment of both physical and mental health symptoms. The provision of care for patients with a mental dysregulation can evoke a wide variety of feelings in ECC staff, ranging from worry or pity, to annoyance and frustration (8,24-27). These feelings may originate from several challenges in the ECC for these patients. First, these challenges may be induced by the still existing and experienced segregation between somatic and mental treatment, long waiting times (28), and revisits of patients with a mental dysregulation which cause irritation and a decrease in confidence in the effectiveness of interventions. Second, healthcare professionals in the ECC do not always have the right knowledge of psychopathology and legislation (21,29) or the skills to use de-escalation techniques in a timely fashion (30,31), which can lead to frustration.

These challenges and feelings may lead to stigma towards patients with a mental dysregulation who seek emergency care (26-29,31,32). Several qualitative studies have revealed that patients with mental problems often feel stigmatized by healthcare professionals in the ECC(15,32-34). A recent scoping review of factors that influence emergency department nurses care for patients with mental illness revealed that the emergency department environment greatly impacts the role of emergency department nurses, that their beliefs and perceptions leads to role conflicts, and the lack of knowledge and confidence impacts their view towards patients with mental illness. Specifically, some nurses reported they avoided patients, judged them as less important and attention seeking (25), which implies stigma towards these group of patients.

In their landmark paper Link & Phelan define stigma as existing when elements of labeling, stereotyping, separating ("us" from "them"), status loss, and discrimination co-occur in a power situation that allows these processes to unfold (35). Thornicroft and colleagues use this definition and elaborate on four different types of stigma of which, for this review, interpersonal stigma (also referred to as 'public stigma') is important. Interpersonal stigma refers to the link between

For peer review only - http://bmjopen.bmj.com/site/about/guidelines.xhtml

stereotypes, negative attitudes (prejudice), and negative behavior (discrimination) towards people with mental health conditions. Three related components are part of interpersonal stigma: knowledge (ignorance or misinformation), attitudes (negative emotional reactions, such as prejudice) and behavior (such as avoidance or rejection) (36).

Various interventions have been developed that impact attitude and behavior and minimize stigma held by health care professionals. Thornicroft and colleagues report on 56 reviews, documenting changes in knowledge, attitudes, and clinical skills as well as clinical confidence and self-efficacy as stigma related outcomes. Several types of interventions are mentioned, such as e-interventions, use of simulations (e.g., standardized role-plays with actors), and social contact, such as filmed or live recovery testimonials. Studies involving different methods of contact (e.g., live or filmed) were more often associated with better outcomes for knowledge and attitudes when compared to educational interventions alone. Interventions including experts with lived experience in the design and evaluation of stigma interventions demonstrated better outcomes (36). The interventions outlined from the reviews covered various populations (e.g., mental health professionals, health care providers) and various mental health problems (e.g., dementia care, suicide prevention, borderline personality disorder); only one publication was specific to emergency department healthcare staff and focused on their attitudes towards patients with substance-related presentations (37). To our knowledge, a comprehensive review of the available scientific evidence in this specific ECC is lacking.

AIM

To review the body of evidence to gain insight into interventions targeting healthcare professionals, that minimize stigma including beliefs, attitudes, and behavior towards patients with a mental dysregulation within the ECC.

CONCEPTS

For this review, we define *patients with a mental dysregulation* in the ECC as follows: patients with mental problems that seek emergency care may experience disproportionate feelings like fear, anger, sadness, or confusion, possibly with associated behaviors. When the aforementioned symptoms interfere with the patient's treatment or that of others, the patient is considered to have a mental dysregulation.

We applied the World Health Organization definition of '*Intervention*', namely, 'an act performed for, with or on behalf of a person or population whose purpose is to assess, improve, maintain, promote, or modify health, functioning or health conditions' (38).

For peer review only - http://bmjopen.bmj.com/site/about/guidelines.xhtml

For peer review only

Stigma is defined as the link between stereotypes, negative attitudes, and discrimination against people with mental health conditions in society (36).

Interpersonal stigma encompasses the association between stereotypes, adverse attitudes (prejudice), and detrimental actions (discrimination) directed at individuals dealing with mental health conditions. It comprises three interconnected elements: knowledge (comprising ignorance or misconceptions), attitudes (comprising unfavorable emotional responses, like prejudice), and behavior (encompassing actions such as avoidance or rejection) (36).

The emergency care context (ECC) in this review is defined as emergency care from the scene of acute incident of illness, followed by ambulance transport to and treatment in the emergency department.

Healthcare professionals include paramedics, emergency care nurses, nurse specialists, physicians and physician assistants that are directly involved in providing emergency care.

RESEARCH QUESTIONS

1. Which interventions that address interpersonal stigma towards patients with a mental dysregulation have been designed, implemented and evaluated for healthcare professionals in the ECC?
2. From the healthcare professionals' perspective, which facilitators and barriers were encountered when implementing these interventions within the ECC?
3. From the service users' perspective, which facilitators and barriers were encountered when implementing these interventions within the ECC?

For peer review only

METHODS AND ANALYSIS

We will perform an integrative review of empirical literature using methodological strategies proposed by Whittemore and Knafl(39), using a 7-step framework outlined by Dhollande and colleagues(40) to identify current state of the art knowledge on the topic and to identify current knowledge gaps in research. The 7-steps applied to the proposed integrative review will involve: 1) write the review question; 2) determine the search strategy; 3) critically appraise the search results; 4) summarize the search results; 5) extract and reduce data; 6) analysis; and 7) conclusions and implications (40). An integrative review allows for inclusion of diverse study methodologies, including qualitative, quantitative, experimental, and mixed-method studies, which makes it suitable for topics that have not been studied intensively. Results from an integrative review provide a complete and varied perspective of the studied topic.

To further enhance the quality of the review, the PRISMA-P recommendations (41) will be followed where applicable(41). The PRISMA-P checklist is attached as Appendix I. Software applications RefWorks-ProQuest, Rayyan and ATLAS.ti will be used to enhance the quality of the review, efficiency of the research work, and transparency of process and results.

Patient and Public Involvement

As Thornicroft and colleagues highlight in their publication on stigma it is important to involve people with lived experience of mental health conditions (PWLE) in both research and innovation projects (36). For that reason, one or two PWLE will be included in the project team and co-author the review. For the development of this review protocol no patient or PWLE was involved.

Data sources and search strategy

A systematic search will be applied, followed by identifying possible missed publications via the reference lists of included publications. The following scientific databases will be systematically searched for English and Dutch publications: CINAHL, PsychINFO and MEDLINE. Search terms will include 'Stigma', 'Interpersonal stigma', 'Public stigma', 'Interventions', 'Emergency healthcare', 'Emergency healthcare staff', 'mental health', emergency department', 'ambulance', 'paramedics' and related search terms, as used in the aforementioned databases. The full search strategy is attached as Appendix II. The results will be presented in the review. Additionally, the grey literature will be searched. This grey literature will be limited to National Guidelines related to this topic (ECC) in English or Dutch. We will search via Google and via Guideline Organizations (e.g., The National Institute for Health and Care Excellence, NICE, in the United Kingdom) and professional organizations (e.g., the American Academy of Emergency Medicine).

For peer review only - http://bmjopen.bmj.com/site/about/guidelines.xhtml

For peer review only

Study selection and sorting

Studies will be included using the following inclusion and exclusion criteria:

Inclusion

- Original research report, both qualitative and quantitative studies;
- RCTs, prospective and retrospective cohort studies, case control studies, cross sectional studies and, if available, literature reviews;
- Description of innovation projects related to the topic; depending on information on outcome variables (see *Data extraction, analysis, and synthesis*-paragraph below);
- Focused on intervention(s), targeting healthcare professionals, to reduce stigma on mental health problems held by ECC staff;
- Peer reviewed publication;
- Published in English or Dutch.
- To obtain as much information as possible on relevant interventions, the search includes thesis and conference abstracts.

Exclusion

- Abstract and/ or full text not available

Study-selection: The results of the search will be assessed in the following steps: 1) Review of title and abstract and 2) Full text review of selected publications. Publications that clearly do not answer the research question or address the inclusion criteria will be excluded (i.e., other topic, not an ECC, no stigma related intervention, language). Both steps will be performed by two independent reviewers, using Rayyan, a web-based tool, designed for publications-selection in scientific literature review (42). When no consensus is reached on a particular publication a third reviewer will make a final decision. A PRISMA flow diagram will be used to provide a summary of the search and selection process.

Quality appraisal

The quality of included studies will be assessed with the Mixed Methods Appraisal Tool (43), developed and improved by Hong and colleagues (44). Each included publication will be assessed by two independent reviewers. Quality appraisal is not used for study selection, but for means of interpreting the strength of findings and conclusions. As Thornicrofft and colleagues (36) emphasize the importance of including persons with lived experience in the development of interventions, we

For peer review only - http://bmjopen.bmj.com/site/about/guidelines.xhtml

will include this in reviewing and appraising interventions presented in the included publications, by answering this question: To what extent are people with lived experience involved in the design/ delivery/ evaluation of the intervention and if involved, what were the results of this?

Data extraction, analysis, and synthesis

Data extraction will be performed using Atlas.ti, a software application, originally designed for qualitative research analyses purposes, but also used for transparent data extraction and analyses in literature review (45). A predetermined set of labels will be set up within ATLAS.ti and will include (but is not limited to): definition of stigma, description of intervention, study population, study design, sample, site/setting, measurements, outcome variable(s), reported strengths of the study, reported limitations of the study.

The main outcome variables of interest are 1) Level of interpersonal stigma and 2) patient satisfaction. The secondary outcome variable of interest is: Level of knowledge on psychiatry.

Depending on the instruments used in the original publications, the measures and instruments for results on the outcome variables will be listed, when appropriate in table(s). In the discussion part of the review, the quality of the used instruments will be discussed.

Two independent reviewers will analyze the included publications, identifying (by adding the predefined labels to) citations in the publications, relevant for answering the review questions. Depending on the content of the results, we will, when appropriate, perform thematic analysis as described by Dhollande and colleagues(40).

The selected citations will be further analyzed and discussed by the authors, using ATLAS.ti tools, whereafter the extracted data will be used for answering the research questions.

Ethics and dissemination

No ethical approval or safety considerations are required for this review. The proposed review will be submitted to a relevant peer reviewed international journal. Results will be presented at relevant medical scientific conferences.

For peer review only - http://bmjopen.bmj.com/site/about/guidelines.xhtml

For peer review only

ADDITIONAL INFORMATION

Planning and time-frame

Start search: Immediately after first submission of the review protocol: February 2023 and repeated in July 2023.

Study selection: February/August 2023

Data extraction and synthesis: April/September 2023

Writing and submission of the review: October/November 2023

Author Statement

GVdG, NG, WHWH developed the initial protocol: review questions, introduction and methods. NB, LS, FS, JC and MvV commented on the manuscript and re-wrote sections of the manuscript. All authors documented their approval of the final version of the review protocol before submission. As senior researcher and project-leader WHWH is the guarantor of the review and review protocol.

Data Statement

As ATLAS.ti is used for data extraction, analysis and synthesis, used citations including used labels from included publications are available via the corresponding author.

Declaration of Conflicting Interests

The authors declare to have no conflicts of interest related to this review.

Funding statement

This review is part of the PONTAC-study (Psychisch ONTregeld in de Acute zorg/ Mental dysregulation in Acute healthcare). The PONTAC study is co-financed by SIA-RAAK, part of the Netherlands Organization for Scientific Research (NWO), under award/grant number: RAAK.PUB08.029. Furthermore, this study was partly funded by a fellowship funding from the Netherlands Organisation for Health Research and Development (ZonMw), under award/grant number number 10040022110004, was awarded to dr. Wietske H.W. Ham (Wietske Blom-Ham).

The University of Applied Sciences, Utrecht, University Medical Centre, Utrecht, Diakonessenhuis, Utrecht, St.Antionius Hospital, Utrecht, Meander Medical Center, Amersfoort, Regional Ambulance

For peer review only - http://bmjopen.bmj.com/site/about/guidelines.xhtml

Service Utrecht, Altrecht, Mental Healthcare, Utrecht have contributed in-kind to the study. The latter 7 institutions did not provide an award/grant number to this project.

For peer review only

For peer review only - http://bmjopen.bmj.com/site/about/guidelines.xhtml

References

(1) Whiteford HA, Ferrari AJ, Degenhardt L, Feigin V, Vos T. The global burden of mental, neurological and substance use disorders: an analysis from the Global Burden of Disease Study 2010. PLoS One 2015;10(2):e0116820.

(2) Arias D, Saxena S, Verguet S. Quantifying the global burden of mental disorders and their economic value. EClinicalMedicine 2022 Sep 28;54:101675.

(3) Boden M, Zimmerman L, Azevedo KJ, Ruzek JI, Gala S, Abdel Magid H,S., et al. Addressing the mental health impact of COVID-19 through population health; PMC7934657. Clin.Psychol.Rev. 2021;85(102006):102006.

(4) Xiong J, Lipsitz O, Nasri F, Lui LMW, Gill H, Phan L, et al. Impact of COVID-19 pandemic on mental health in the general population: A systematic review; PMC7413844. J.Affect.Disord. 2020;277:55-64.

(5) Kessler RC, Aguilar-Gaxiola S, Alonso J, Chatterji S, Lee S, Ormel J, et al. The global burden of mental disorders: an update from the WHO World Mental Health (WMH) surveys; PMC3039289. Epidemiol.Psichiatr.Soc. 2009;18(1):23-33.

(6) Scott KM, Lim C, Al-Hamzawi A, Alonso J, Bruffaerts R, Caldas-de-Almeida JM, et al. Association of mental disorders with subsequent chronic physical conditions: World mental health surveys from 17 countries; PMC5333921. JAMA Psychiatry 2016;73(2):150-158.

(7) Momen NC, Plana-Ripoll O, Agerbo E, Benros ME, Børglum A,D., Christensen MK, et al. Association between mental disorders and subsequent medical conditions; PMC7261506. N.Engl.J.Med. 2020;382(18):1721-1731.

(8) Giandinoto J, Edward K. Challenges in acute care of people with co-morbid mental illness. Br J Nurs 2014 Jul 10-23;23(13):728-732.

(9) Giandinoto J, Edward K. The phenomenon of co-morbid physical and mental illness in acute medical care: the lived experience of Australian health professionals. BMC Res Notes 2015 Jul 07,;8:295.

(10) Theriault KM, Rosenheck RA, Rhee TG. Increasing Emergency Department Visits for Mental Health Conditions in the United States. J Clin Psychiatry 2020 Jul 28,;81(5).

(11) Ronaldson A, Elton L, Jayakumar S, Jieman A, Halvorsrud K, Bhui K. Severe mental illness and health service utilisation for nonpsychiatric medical disorders: A systematic review and meta-analysis. PLoS Med 2020 09;17(9):e1003284.

(12) Liu NH, Daumit GL, Dua T, Aquila R, Charlson F, Cuijpers P, et al. Excess mortality in persons with severe mental disorders: a multilevel intervention framework and priorities for clinical practice, policy and research agendas; PMC5269481. World Psychiatry 2017;16(1):30-40.

(13) Azar KMJ, Petersen JP, Shen Z, Nasrallah C, Pesa J, LaMori J, et al. Serious Mental Illness and Health-Related Factors Associated with Regional Emergency Department Utilization. Popul Health Manag 2020 Dec;23(6):430-437.

For peer review only

For peer review only - http://bmjopen.bmj.com/site/about/guidelines.xhtml

(14) Armoon B, Cao Z, Grenier G, Meng X, Fleury M. Profiles of high emergency department users with mental disorders. Am J Emerg Med 2022;54:131-141.

(15) Sacre M, Albert R, Hoe J. What are the experiences and the perceptions of service users attending Emergency Department for a mental health crisis? A systematic review. Int.J.Ment.Health Nurs. 2022;31(2):400-423.

(16) Artioli M, Bougon E, Mathur A, Salles J. Factors associated with a borderline personality disorder diagnosis in the emergency department. Front Psychiatry 2022;13:925462.

(17) Dowdell EB, Alderman SE, Foushee N, Holland E, Reedy EA. Expressions of Compassion Fatigue by Emergency Department Nurses Caring for Patients With Opioid and Substance Use Disorders. J Emerg Nurs 2022;48(6):688-697.

(18) Rassy J, Daneau D, Larue C, Rahme E, Low N, Lamarre S, et al. Measuring Quality of Care Received by Suicide Attempters in the Emergency Department. Arch Suicide Res 2022;26(1):81-90.

(19) Innes K, Morphet J, O'Brien AP, Munro I. Caring for the mental illness patient in emergency departments--an exploration of the issues from a healthcare provider perspective. J Clin Nurs 2014 Jul;23(13-14):2003-2011.

(20) van Nieuwenhuizen A, Henderson C, Kassam A, Graham T, Murray J, Howard LM, et al. Emergency department staff views and experiences on diagnostic overshadowing related to people with mental illness. Epidemiol Psychiatr Sci 2013 Sep;22(3):255-262.

(21) Marynowski-Traczyk D, Moxham L, Broadbent M. Emergency Department Registered Nurses' conceptualisation of recovery for people experiencing mental illness. Australas Emerg Nurs J 2017 May;20(2):75-81.

(22) Rolfe U, Pope C, Crouch R. Paramedic performance when managing patients experiencing mental health issues - Exploring paramedics' Presentation of Self. Int Emerg Nurs 2020 03;49:100828.

(23) Shefer G, Henderson C, Howard LM, Murray J, Thornicroft G. Diagnostic overshadowing and other challenges involved in the diagnostic process of patients with mental illness who present in emergency departments with physical symptoms--a qualitative study. PLoS One 2014;9(11):e111682.

(24) Beks H, Healey C, Schlicht KG. 'When you're it': a qualitative study exploring the rural nurse experience of managing acute mental health presentations. Rural Remote Health 2018 08;18(3):4616.

(25) Perrone McIntosh J,T. Emergency department nurses' care of psychiatric patients: A scoping review. Int.Emerg.Nurs. 2021;54(100929):100929.

(26) Clarke D, Usick R, Sanderson A, Giles-Smith L, Baker J. Emergency department staff attitudes towards mental health consumers: a literature review and thematic content analysis. Int J Ment Health Nurs 2014 Jun;23(3):273-284.

(27) Emond K, O'Meara P, Bish M. Paramedic management of mental health related presentations: a scoping review. J Ment Health 2019 Feb;28(1):89-96.

For peer review only - http://bmjopen.bmj.com/site/about/guidelines.xhtml

(28) Sweeny A, Keijzers G, O'Dwyer J, Stapelberg NC, Crilly J. Patients with mental health conditions in the emergency department: Why so long a wait? Emerg Med Australas 2020 Jun 08,.

(29) Mérelle SYM, Boerema I, van der Linden MC, Gilissen R. [Issues in emergency care for people who attempted suicide]. Ned Tijdschr Geneeskd 2018 Jul 02,;162.

(30) Rolfe U, Pope C, Crouch R. Paramedic performance when managing patients experiencing mental health issues - Exploring paramedics' Presentation of Self. Int Emerg Nurs 2020 03;49:100828.

(31) McCann TV, Savic M, Ferguson N, Cheetham A, Witt K, Emond K, et al. Recognition of, and attitudes towards, people with depression and psychosis with/without alcohol and other drug problems: results from a national survey of Australian paramedics. BMJ Open 2018 12 04,;8(12):e023860.

(32) Wong AH, Ray JM, Rosenberg A, Crispino L, Parker J, McVaney C, et al. Experiences of Individuals Who Were Physically Restrained in the Emergency Department. JAMA Netw Open 2020 01 03,;3(1):e1919381.

(33) Daggenvoorde TH, Gijsman HJ, Goossens PJJ. Emergency care in case of acute psychotic and/or manic symptoms: Lived experiences of patients and their families with the first interventions of a mobile crisis team. A phenomenological study. Perspect Psychiatr Care 2018 Oct;54(4):462-468.

(34) Digel Vandyk A, Young L, MacPhee C, Gillis K. Exploring the Experiences of Persons Who Frequently Visit the Emergency Department for Mental Health-Related Reasons. Qual Health Res 2018 03;28(4):587-599.

(35) Link, Bruce G. Phelan, Jo C. Conceptualizing Stigma. . annurev.soc. 2001;27:363-385.

(36) Thornicroft G, Sunkel C, Alikhon Aliev A, Baker S, Brohan E, El Chammay R, et al. The Lancet Commission on ending stigma and discrimination in mental health. Lancet 2022;400(10361):1438-1480.

(37) Gonzalez M, Clarke DE, Pereira A, Boyce-Gaudreau K, Waldman C, Demczuk L, et al. The impact of educational interventions on attitudes of emergency department staff towards patients with substance-related presentations: a quantitative systematic review. JBI Database System Rev Implement Rep 2017 Aug;15(8):2153-2181.

(38) Fortune N, Madden R, Almborg A. Use of a new international classification of Health Interventions for capturing information on Health Interventions relevant to people with disabilities; PMC5800244. Int.J.Environ.Res.Public Health 2018;15(1).

(39) Whittemore R, Knafl K. The integrative review: updated methodology. J.Adv.Nurs. 2005;52(5):546-553.

(40) Dhollande S, Taylor A, Meyer S, Scott M. Conducting integrative reviews: a guide for novice nursing researchers. J Res Nurs 2021 Aug;26(5):427-438.

(41) Shamseer L, Moher D, Clarke M, Ghersi D, Liberati A, Petticrew M, et al. Preferred reporting items for systematic review and meta-analysis protocols (PRISMA-P) 2015: elaboration and explanation. BMJ 2015 Jan 2;350:g7647.

For peer review only - http://bmjopen.bmj.com/site/about/guidelines.xhtml

(42) Rayyan. Faster systematic reviews. 2023; Available at: https://www.rayyan.ai/. Accessed 03-21-, 2023.

(43) Mixed Methods Appraisal Tool - version 2018. 2018; Available at: http://mixedmethodsappraisaltoolpublic.pbworks.com/w/file/fetch/127916259/MMAT_2018_criteria-manual_2018-08-01_ENG.pdf. Accessed 20th December, 2022.

(44) Hong QN, Gonzalez-Reyes A, Pluye P. Improving the usefulness of a tool for appraising the quality of qualitative, quantitative and mixed methods studies, the Mixed Methods Appraisal Tool (MMAT). J.Eval.Clin.Pract. 2018;24(3):459-467.

(45) Smit B, Scherman V. Computer-Assisted Qualitative Data Analysis Software for Scoping Reviews: A Case of ATLAS.ti. International Journal of Qualitative Methods 2021;20:16094069211019140.

For peer review only

For peer review only - http://bmjopen.bmj.com/site/about/guidelines.xhtml

Appendix I: PRISMA-P checklist

Section and topic	Item No	Checklist item	How and where is the item addressed in this review protocol
**ADMINISTRATIVE INFORMATION**			
Title:			
Identification	1a	Identify the report as a protocol of a systematic review	the title (p1) and methods (p7) describe the review as an integrative review.
Update	1b	If the protocol is for an update of a previous systematic review, identify as such	Not applicable
Registration	2	If registered, provide the name of the registry (such as PROSPERO) and registration number	PROSPERO ID number: CRD42023390664 Added to the abstract (p2)
Authors:			
Contact	3a	Provide name, institutional affiliation, e-mail address of all protocol authors; provide physical mailing address of corresponding author	Name, affiliations of all protocol authors are listed. Email address and physical mailing address of the corresponding author is provided on the title page. E-mail addresses of co-authors are presented to the publisher of the review protocol.
Contributions	3b	Describe contributions of protocol authors and identify the guarantor of the review	GVdG, NG, WHWH developed the initial protocol: review questions, introduction and methods. NB, LS, FS, JC, MvV commented on the manuscript and re-wrote sections of the manuscript. All authors documented their approval of the final version of the review protocol before submission. As senior researcher and project-leader WHWH is the guarantor of the review and review protocol.<br>This information can be found in the 'Author Statement' (p10).
Amendments	4	If the protocol represents an amendment of a previously completed or published protocol, identify as such and list changes; otherwise, state plan for documenting important protocol amendments	n.a.
Support:			
Sources	5a	Indicate sources of financial or other support for the review	Provided in the section Funding Statement (p10-11)
Sponsor	5b	Provide name for the review funder and/or sponsor	Provided in the section Funding Statement (p10-11)
Role of sponsor or funder	5c	Describe roles of funder(s), sponsor(s), and/or institution(s), if any, in developing the protocol	The funders/institutions have no role in developing the protocol. The authors, though working for some of these institutions, work independently on this research.
**INTRODUCTION**			
Rationale	6	Describe the rationale for the review in the context of what is already known	Provided in the introduction (p3-5)

For peer review only

Objectives	7	Provide an explicit statement of the question(s) the review will address with reference to participants, interventions, comparators, and outcomes (PICO)	Participants/Interventions: Provided in the Aim/Concepts/Research questions/ Methods and Analyses sections (p5-7)<br>Comparators will be documented, dependent on what is given in original publications.<br>Outcome variables: Main outcome variables: 1)Level of Interpersonal Stigma and 2) Patient satisfaction. The secondary outcome variable looked at is: Level of knowledge on psychiatry (p9).
**METHODS**			
Eligibility criteria	8	Specify the study characteristics (such as PICO, study design, setting, time frame) and report characteristics (such as years considered, language, publication status) to be used as criteria for eligibility for the review	All addressed in the protocol, in the 'Study selection and sorting'- section (p8).
Information sources	9	Describe all intended information sources (such as electronic databases, contact with study authors, trial registers or other grey literature sources) with planned dates of coverage	Listed in the Methods section: Medline, PsychInfo, CINAHL (p7).<br>Grey literature: Search via Google and contacting Professional Organizations for National Guidelines on the topic, in English and Dutch speaking countries (p7).
Search strategy	10	Present draft of search strategy to be used for at least one electronic database, including planned limits, such that it could be repeated	The full search strategy is attached as Appendix II to this review protocol.
Study records:			
Data management	11a	Describe the mechanism(s) that will be used to manage records and data throughout the review	Refworks Proquest will be used for management of the records.<br>Rayyan will be used for independent selection of studies, by two independent reviewers. Atlas.ti.23 will be used for data-extraction (p7).
Selection process	11b	State the process that will be used for selecting studies (such as two independent reviewers) through each phase of the review (that is, screening, eligibility and inclusion in meta-analysis)	Addressed in the 'Study selection and sorting' section. All steps in screening, in- and exclusion process, data-extraction and appraisal is carried out by two independent reviewers (p8).
Data collection process	11c	Describe planned method of extracting data from reports (such as piloting forms, done independently, in duplicate), any processes for obtaining and confirming data from investigators	Addressed in the 'Data extraction, analysis, and synthesis' section. Data extraction to be performed by two independent reviewers. Using ATLAS.ti (p9).
Data items	12	List and define all variables for which data will be sought (such as PICO items, funding sources), any pre-planned data assumptions and simplifications	Addressed in the 'Concepts' section (p5-6).
Outcomes and prioritization	13	List and define all outcomes for which data will be sought, including prioritization of main and additional outcomes, with rationale	Provided in the 'Data extraction, analysis, and synthesis' section (p9).

For peer review only

Risk of bias in individual studies	14	Describe anticipated methods for assessing risk of bias of individual studies, including whether this will be done at the outcome or study level, or both; state how this information will be used in data synthesis	Addressed in the 'Quality appraisal' section. Each included publication will be rated using the MMAT by two independent reviewers (p8-9).
Data synthesis	15a	Describe criteria under which study data will be quantitatively synthesized	a. and b. Given the nature of the outcome variables, and the expected paucity in available original research, we expect not to be able to quantitatively synthesize results.
	15b	If data are appropriate for quantitative synthesis, describe planned summary measures, methods of handling data and methods of combining data from studies, including any planned exploration of consistency (such as I^2, Kendall's τ)	Included studies will be listed in tables, presenting the main characteristics of the included studies: type of innovation, level of development, outcome measures, type of evaluation/testing of the innovations.
	15c	Describe any proposed additional analyses (such as sensitivity or subgroup analyses, meta-regression)	For each of the included studies, the results of the quality appraisal, using the MMAT (Mixed Methods Appraisal Tool) will be documented.
	15d	If quantitative synthesis is not appropriate, describe the type of summary planned	
Meta-bias(es)	16	Specify any planned assessment of meta-bias(es) (such as publication bias across studies, selective reporting within studies)	Given the expected paucity in available original research we do not expect Meta-bias(es).
Confidence in cumulative evidence	17	Describe how the strength of the body of evidence will be assessed (such as GRADE)	We will use the MMAT results as an indication for the overall quality of included publications. If the number and quality of included publications exceeds expectations, the authors will decide on using GRADE as an instrument for grading the quality of the included research (p8-9).

For peer review only

Appendix II: Search Strategy

Date Search: 13th July 2023

Medline/Pubmed:

Search	Query
#1	Attitude of Health Personnel[Mesh] OR Social Stigma[Mesh] OR Professional Competence[Mesh] OR “Professional-Patient Relations”[Mesh] OR “Social Discrimination”[Mesh] OR Attitude*[tiab] OR Stigma[tiab] OR Competence[tiab] OR “Professional Patient Relation*”[tiab] OR Discrimination[tiab] OR Traumatization[tiab] OR Traumatisation[tiab] OR “Secondary Trauma*”[tiab]
#2	Emergency Nursing[Mesh] OR "Emergency Nurs*"[tiab] OR "Emergency Room Nurs*"[tiab] OR "Trauma Nursing"[tiab] OR Emergency Medical Services[Mesh] OR Hospital Emergency Service[Mesh] OR "Emergency Medical Service*"[tiab] OR "Emergency Care"[tiab] OR "Emergency Health Service*"[tiab] OR "Emergency Department*"[tiab] OR "Emergency Room*"[tiab] OR "Emergency Unit*"[tiab] OR "Emergency Ward*"[tiab] OR "Emergency Hospital*"[tiab] OR "Emergency Service*"[tiab] OR Paramedics[Mesh] OR Paramedic*[tiab] OR "Emergency Physician*"[tiab] OR Ambulance*[tiab] OR "First Responder*"[tiab]
#3	Mental Disorders[Mesh] OR "Mental Disorder*"[tiab] OR "Mental Health Issue*"[tiab] OR "Mental Illness*"[tiab] OR "Mental Health Presentation*"[tiab] OR "Mental Health Problem*"[tiab] OR "Psychiatric Disorder*"[tiab] OR "Psychiatric Disease*"[tiab] OR "Psychiatric Illness*"[tiab] OR "Psychiatric Condition*"[tiab] OR Psychophysiologic Disorders[Mesh] OR "Psychophysiologic Disorder*"[tiab] OR "Psychosomatic Disorder*"[tiab] OR Aggression[Mesh] OR Aggression*[tiab] OR Problem Behavior[Mesh] OR "Problem Behavior*"[tiab] OR "Behavioral Problem*"[tiab] OR "Disruptive Behavior*"[tiab] OR "Behavioral Disorder*"[tiab] OR "Psychologically disturbed"[tiab] OR Self-Injurious Behavior[Mesh] OR "Self Injur*"[tiab] OR "Self Harm"[tiab] OR "Self Destruct*"[tiab] OR "Self Mutilat*"[tiab] OR Suicide[tiab] OR Suicidal[tiab]
#4	Intervention*[tiab] OR Action*[tiab] OR Educat*[tiab] OR Knowledge[tiab] OR Consult*[tiab] OR Training[tiab] OR Course*[tiab] OR Treatment[tiab] OR Therap*[tiab] OR Medication[tiab] OR Protocol*[tiab] OR Instrument*[tiab] OR Tool*[tiab]
#5	#1 AND #2 AND #3 AND #4

For peer review only

CINAHL:

Search	Query
#1	MH ("Attitude of Health Personnel+" OR "Professional Competence+" OR Stigma OR "Professional-Patient Relations+" OR Discrimination+) OR TI (Attitude* OR Stigma OR Competence OR "Professional Patient Relation*"OR Discrimination OR Traumatization OR Traumatisation OR "Secondary Trauma*") OR AB (Attitude* OR Stigma OR Competence OR "Professional Patient Relation*" OR Discrimination OR Traumatization OR Traumatisation OR "Secondary Trauma*")
#2	MH ("Emergency Medical Services+" OR "Emergency Nursing+" OR "Emergency Medical Technicians") OR TI ("Emergency Nurs*" OR "Emergency Room Nurs*" OR "Trauma Nursing" OR "Emergency Medical Service*" OR "Emergency Care" OR "Emergency Health Service*" OR "Emergency Department*" OR "Emergency Room*" OR "Emergency Unit*" OR "Emergency Ward*" OR "Emergency Hospital*" OR "Emergency Service*" OR Paramedic* OR "Emergency Physician*" OR Ambulance* OR "First Responder*") OR AB ("Emergency Nurs*" OR "Emergency Room Nurs*" OR "Trauma Nursing" OR "Emergency Medical Service*" OR "Emergency Care" OR "Emergency Health Service*" OR "Emergency Department*" OR "Emergency Room*" OR "Emergency Unit*" OR "Emergency Ward*" OR "Emergency Hospital*" OR "Emergency Service*" OR Paramedic* OR "Emergency Physician*" OR Ambulance* OR "First Responder*")
#3	(MH ("Mental Disorders+" OR "Suicide+" OR "Self-Injurious Behavior") OR TI ("Mental Disorder*" OR "Mental Health Issue*" OR "Mental Illness*" OR "Mental Health Presentation*" OR "Psychiatric Disorder*" OR "Psychiatric Disease*" OR "Psychiatric Illness*" OR "Psychiatric Condition*" OR "Psychophysiologic Disorder*" OR "Psychosomatic Disorder*" OR Aggression* OR Aggressive OR "Problem Behavior*" OR "Behavioral Problem*" OR "Behavior Problem*" OR "Behavior Disorder*" OR "Disruptive Behavior*" OR "Behavioral Disorder*" OR "Psychologically disturbed" OR "Self Injur*" OR "Self Harm" OR "Self Destruct*" OR "Self Mutilat*" OR Suicide OR Suicidal) OR AB ("Mental Disorder*" OR "Mental Health Issue*" OR "Mental Illness*" OR "Mental Health Presentation*" OR "Psychiatric Disorder*" OR "Psychiatric Disease*" OR "Psychiatric Illness*" OR "Psychiatric Condition*" OR "Psychophysiologic Disorder*" OR "Psychosomatic Disorder*" OR Aggression* OR Aggressive OR "Problem Behavior*" OR "Behavioral Problem*" OR "Behavior Problem*" OR "Behavior Disorder*" OR "Psychologically disturbed" OR "Self Injur*" OR "Self Harm" OR "Self Destruct*" OR "Self Mutilat*" OR Suicide OR Suicidal)

For peer review only

#4	TI(Intervention* OR Action* OR Educat* OR Knowledge OR Consult* OR Training OR Course* OR Treatment OR Therap* OR Medication OR Protocol* OR Instrument* OR Tool*) OR AB(Intervention* OR Action* OR Educat* OR Knowledge OR Consult* OR Training OR Course* OR Treatment OR Therap* OR Medication OR Protocol* OR Instrument* OR Tool*)
#5	#1 AND #2 AND #3 AND #4

PsycINFO:

Search	Query
#1	DE (Stigma OR "Health Personnel Attitudes" OR "Professional Competence+" OR Discrimination+) OR TI (Attitude* OR Stigma OR Competence OR "Professional Patient Relation*" OR Discrimination OR Traumatization OR Traumatisation OR "Secondary Trauma*") OR AB (Attitude* OR Stigma OR Competence OR "Professional Patient Relation*" OR Discrimination OR Traumatization OR Traumatisation OR "Secondary Trauma*")
#2	DE ("Emergency Services+" OR "Emergency Personnel+") OR TI ("Emergency Nurs*" OR "Emergency Room Nurs*" OR "Trauma Nursing" OR "Emergency Medical Service*" OR "Emergency Care" OR "Emergency Health Service*" OR "Emergency Department*" OR "Emergency Room*" OR "Emergency Unit*" OR "Emergency Ward*" OR "Emergency Hospital*" OR "Emergency Service*" OR Paramedic* OR "Emergency Physician*" OR Ambulance* OR "First Responder*") OR AB ("Emergency Nurs*" OR "Emergency Room Nurs*" OR "Trauma Nursing" OR "Emergency Medical Service*" OR "Emergency Care" OR "Emergency Health Service*" OR "Emergency Department*" OR "Emergency Room*" OR "Emergency Unit*" OR "Emergency Ward*" OR "Emergency Hospital*" OR "Emergency Service*" OR Paramedic* OR "Emergency Physician*" OR Ambulance* OR "First Responder*")
#3	((DE ("Mental Disorders+" OR "Behavior Disorders+" OR "Behavior Problems+" OR "Aggressive Behavior+" OR "Self-Destructive Behavior+") OR TI ("Mental Disorder*" OR "Mental Health Issue*" OR "Mental Illness*" OR "Mental Health Presentation*" OR "Psychiatric Disorder*" OR "Psychiatric Disease*" OR "Psychiatric Illness*" OR "Psychophysiologic Disorder*" OR "Psychosomatic Disorder*" OR Aggression* OR Aggressive OR "Problem Behavior*" OR "Behavioral Problem*" OR "Behavior Problem*" OR "Behavior Disorder*" OR "Disruptive Behavior*" OR "Behavioral Disorder*" OR "Psychologically disturbed" OR "Self Injur*" OR "Self Harm" OR "Self Destruct*" OR "Self Mutilat*" OR Suicide OR Suicidal) OR AB ("Mental Disorder*" OR "Mental Health Issue*" OR "Mental Illness*" OR "Mental Health Presentation*" OR "Psychiatric Disorder*" OR "Psychiatric Disease*" OR "Psychiatric Illness*" OR

For peer review only

	“Psychophysiologic Disorder*” OR “Psychosomatic Disorder*” OR Aggression* OR Aggressive OR “Problem Behavior*” OR “Behavioral Problem*” OR “Behavior Problem*” OR “Behavior Disorder*” OR “Disruptive Behavior*” OR “Behavioral Disorder*” OR “Psychologically disturbed” OR “Self Injur*” OR “Self Harm” OR “Self Destruct*” OR “Self Mutilat*” OR Suicide OR Suicidal))
#4	TI(Intervention* OR Action* OR Educat* OR Knowledge OR Consult* OR Training OR Course* OR Treatment OR Therap* OR Medication OR Protocol* OR Instrument* OR Tool*) OR AB(Intervention* OR Action* OR Educat* OR Knowledge OR Consult* OR Training OR Course* OR Treatment OR Therap* OR Medication OR Protocol* OR Instrument* OR Tool*)
#5	#1 AND #2 AND #3 AND #4

For peer review only
